# Supplementary material for: Synthesis, Biological Evaluation, and In Silico Studies of Novel Aminated Xanthones as Potential p53-Activating Agents
Source: Molecules. 2019 May 22;24(10):1975. doi: 10.3390/molecules24101975 (PMC6571851; doi:10.3390/molecules24101975)
Supplement: Supplementary file 1 [file molecules-24-01975-s001.pdf]

SUPPLEMENTARY INFORMATION

Synthesis, biological evaluation and *in silico* studies of novel aminated xanthones as potential p53-activating agents

Agostinho Lemos [1], Ana Sara Gomes [2], Joana B. Loureiro [2], Pedro Brandão [1], Andreia Palmeira [1,4], Madalena M. M. Pinto [1,3], Lucília Saraiva [2,*], and Maria Emília Sousa [1,3,*]

[1] Laboratory of Organic and Pharmaceutical Chemistry, Department of Chemical Sciences, Faculty of Pharmacy, University of Porto, Rua de Jorge Viterbo Ferreira, 228, 4050-313 Porto, Portugal; up201002662@ff.up.pt (A.L.); pedrocgbrandao@gmail.com (P.B.); andreiapalmeira@gmail.com (A.P.); madalena@ff.up.pt (M.M.M.P.)

[2] UCIBIO/REQUIMTE, Laboratory of Microbiology, Department of Biological Sciences, Faculty of Pharmacy, University of Porto, Rua de Jorge Viterbo Ferreira, 228, 4050-313 Porto, Portugal; anasarag4@gmail.com (A.S.G.); up201407524@ff.up.pt (J.B.L.)

[3] CIIMAR-Interdisciplinary Centre of Marine and Environmental Research, University of Porto, Novo Edificio do Terminal de Cruzeiros do Porto de Leixões, Avenida General Norton de Matos, S/N, 4450-208 Matosinhos, Portugal.

* Correspondence: esousa@ff.up.pt (E.S.); lucilia.saraiva@ff.up.pt (L.S.); Tel.: +351-22-0428-689 (E.S.); +351-22-0428-584 (L.S.)

1. **HRMS spectra of the described xanthone derivatives**

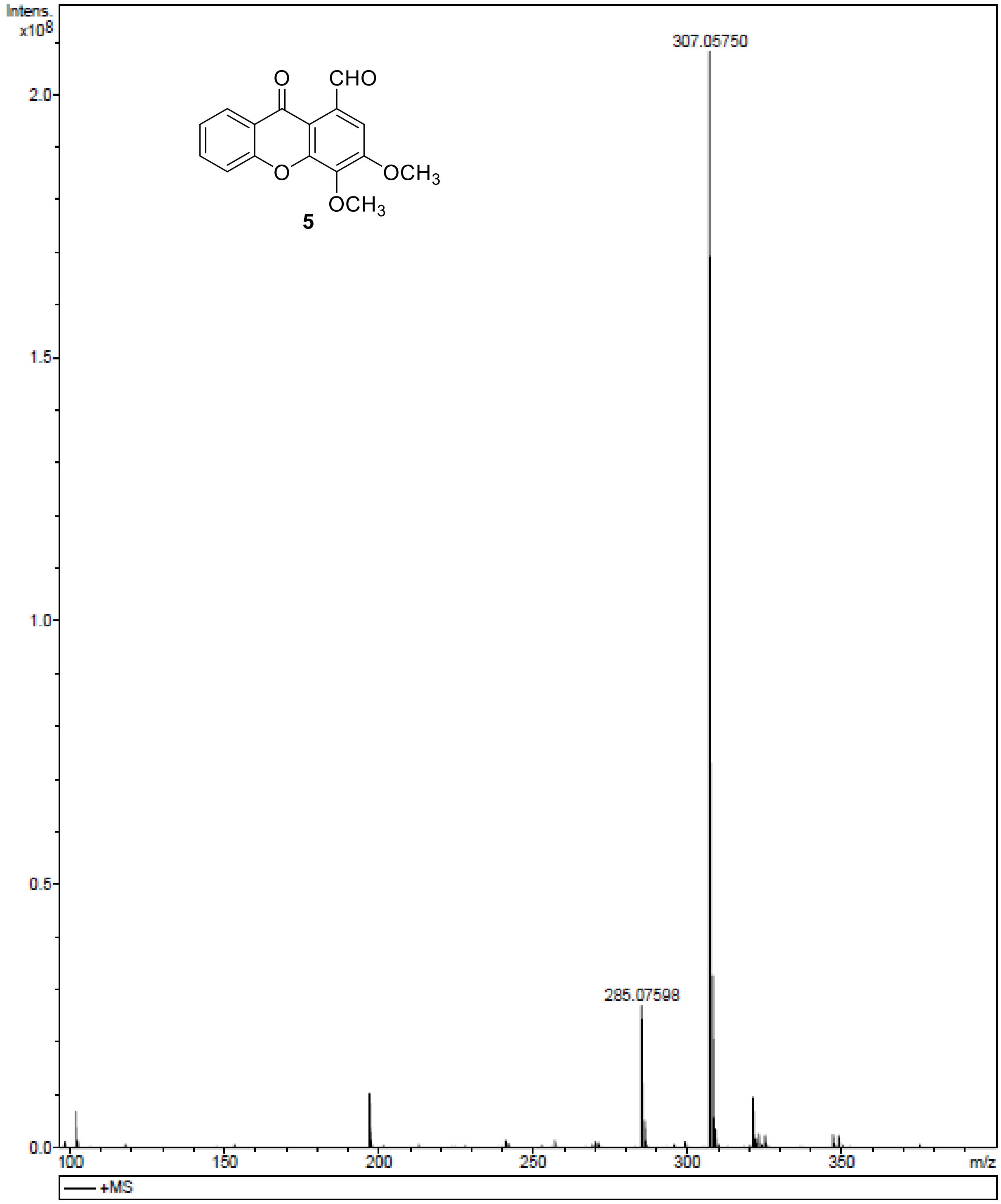

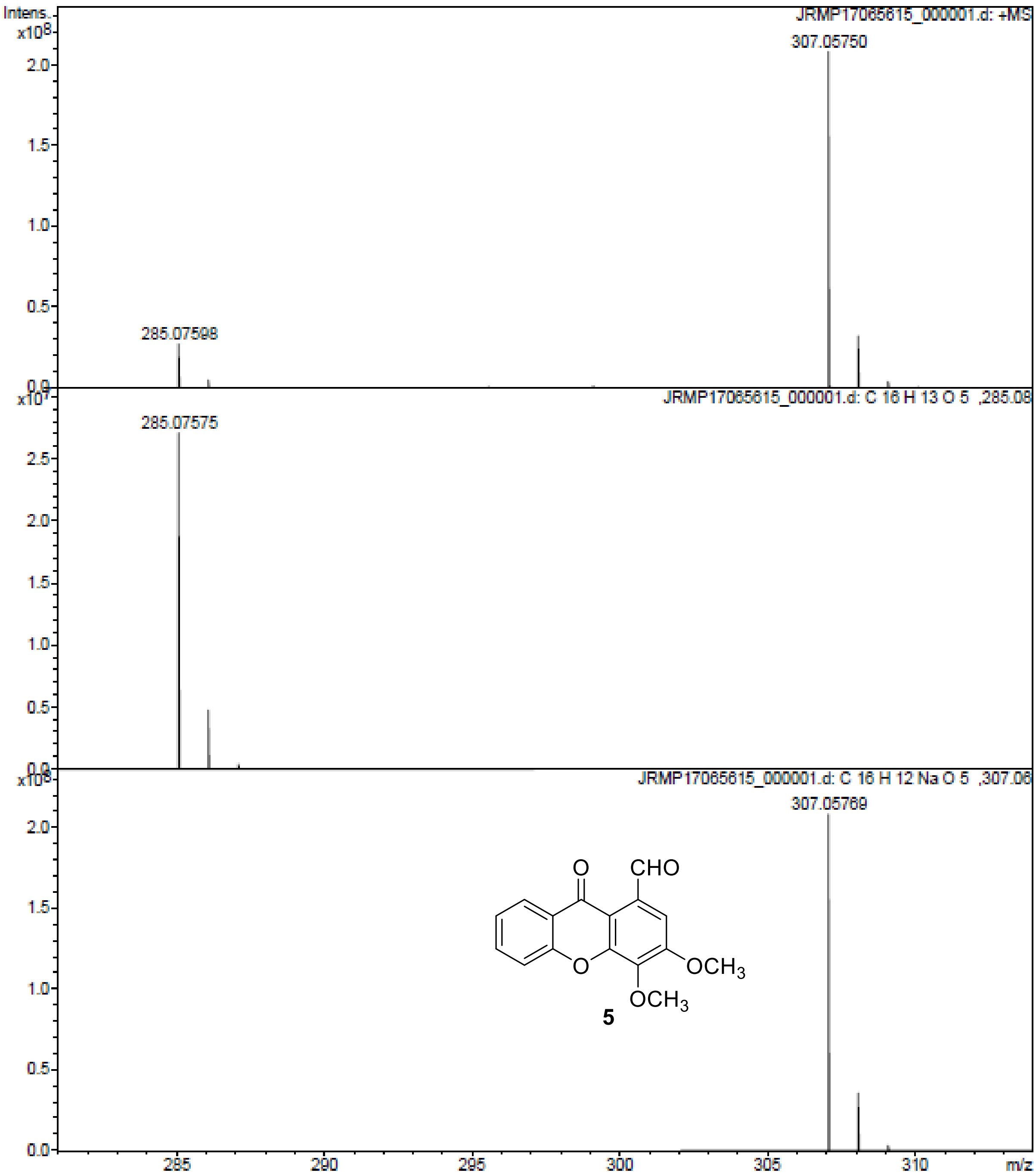

Figure S1. HRMS spectrum of 3,4-dimethoxy-9-oxo-9*H*-xanthene-1-carbaldehyde (**5**).

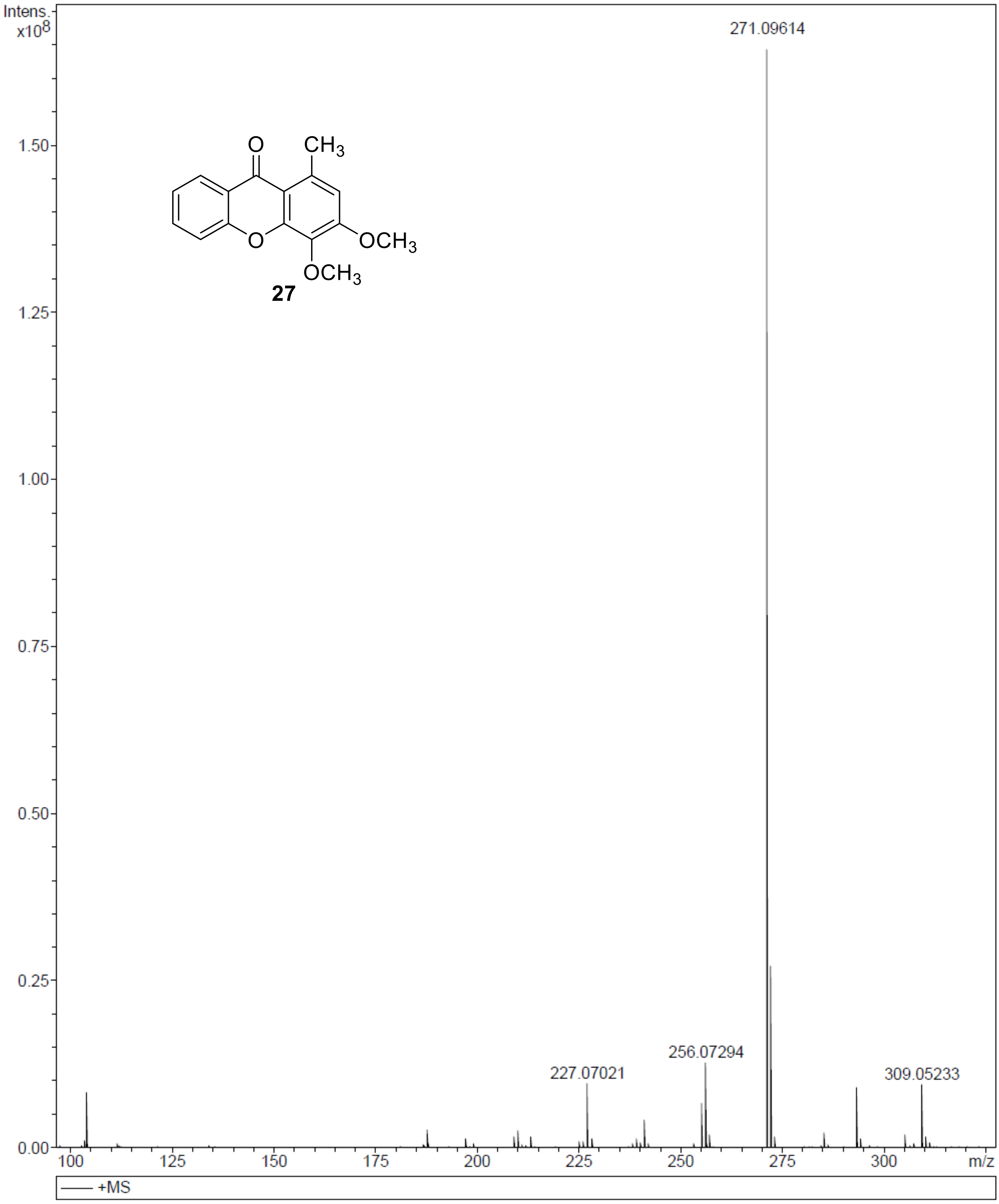
Intens.
x10^8
1.50
1.25
1.00
0.75
0.50
0.25
0.00
271.09614
256.07294
227.07021
309.05233
100
125
150
175
200
225
250
275
300
m/z
+MS
O
CH3
OCH3
OCH3
O
27

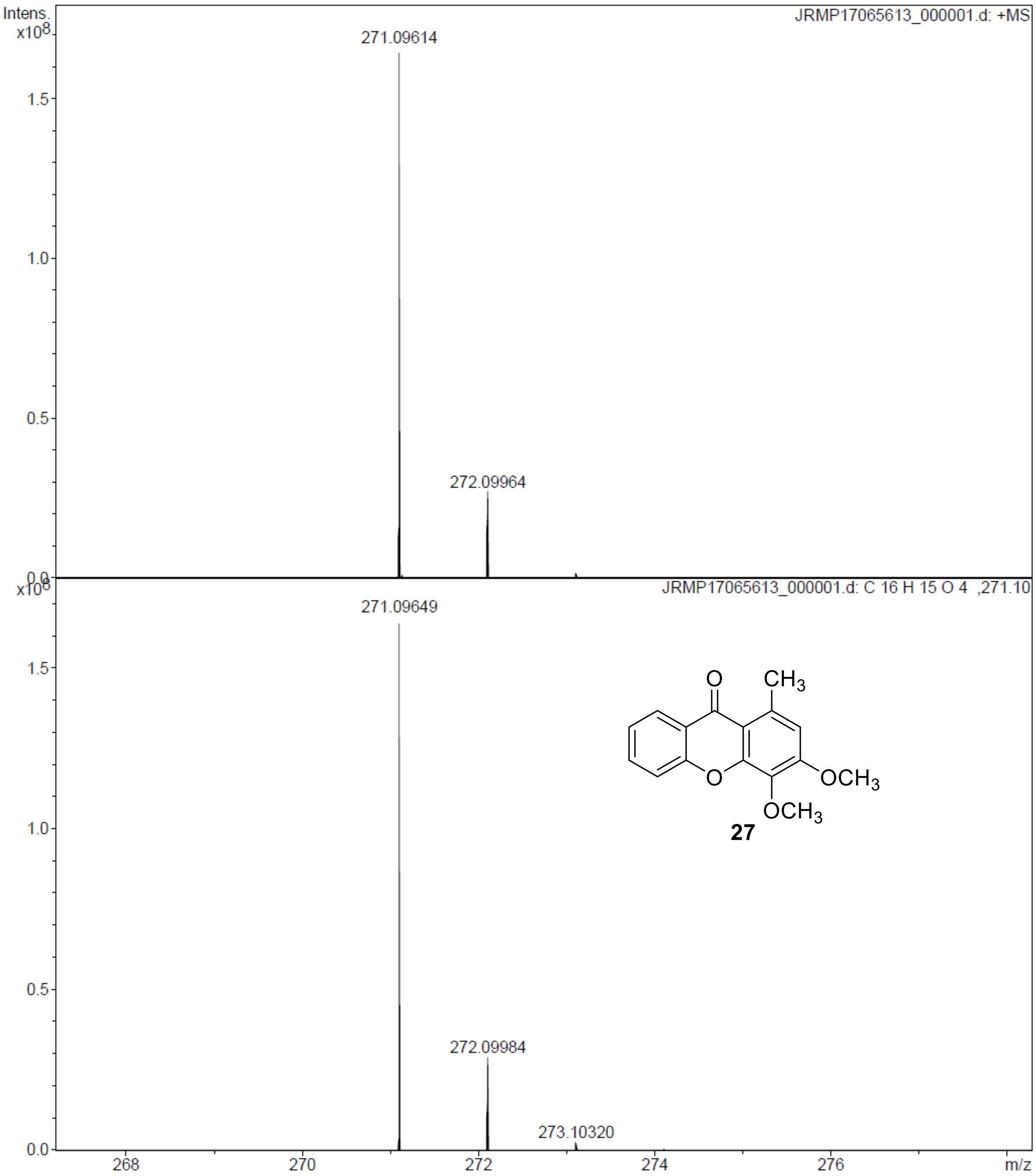

Figure S2. HRMS spectrum of 3,4-dimethoxy-1-methyl-9*H*-xanthen-9-one (**27**).

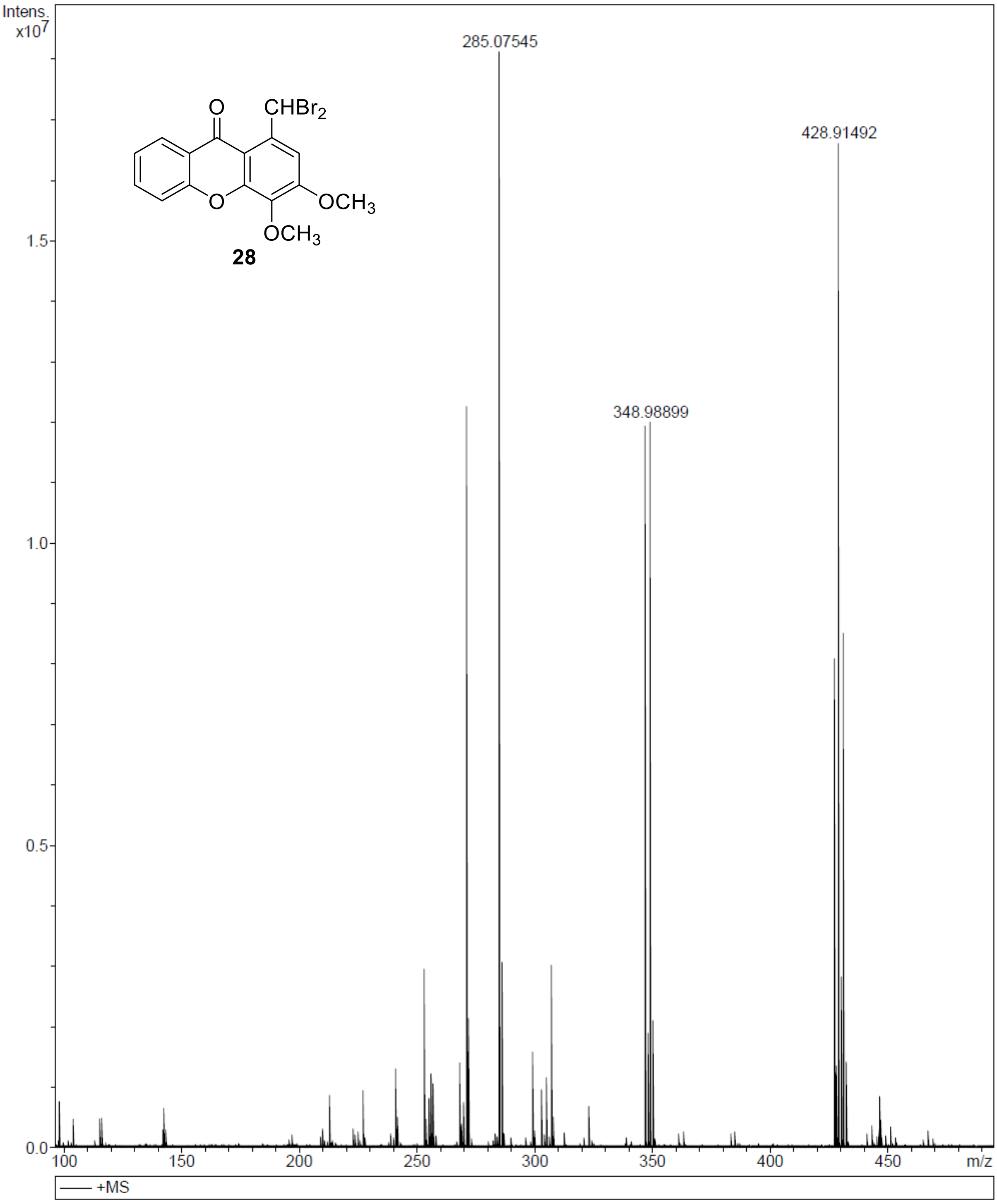

Intens.
x10^7
285.07545
428.91492
348.98899
CHBr2
O
OCH3
OCH3
28
1.5
1.0
0.5
0.0
100
150
200
250
300
350
400
450
m/z
+MS

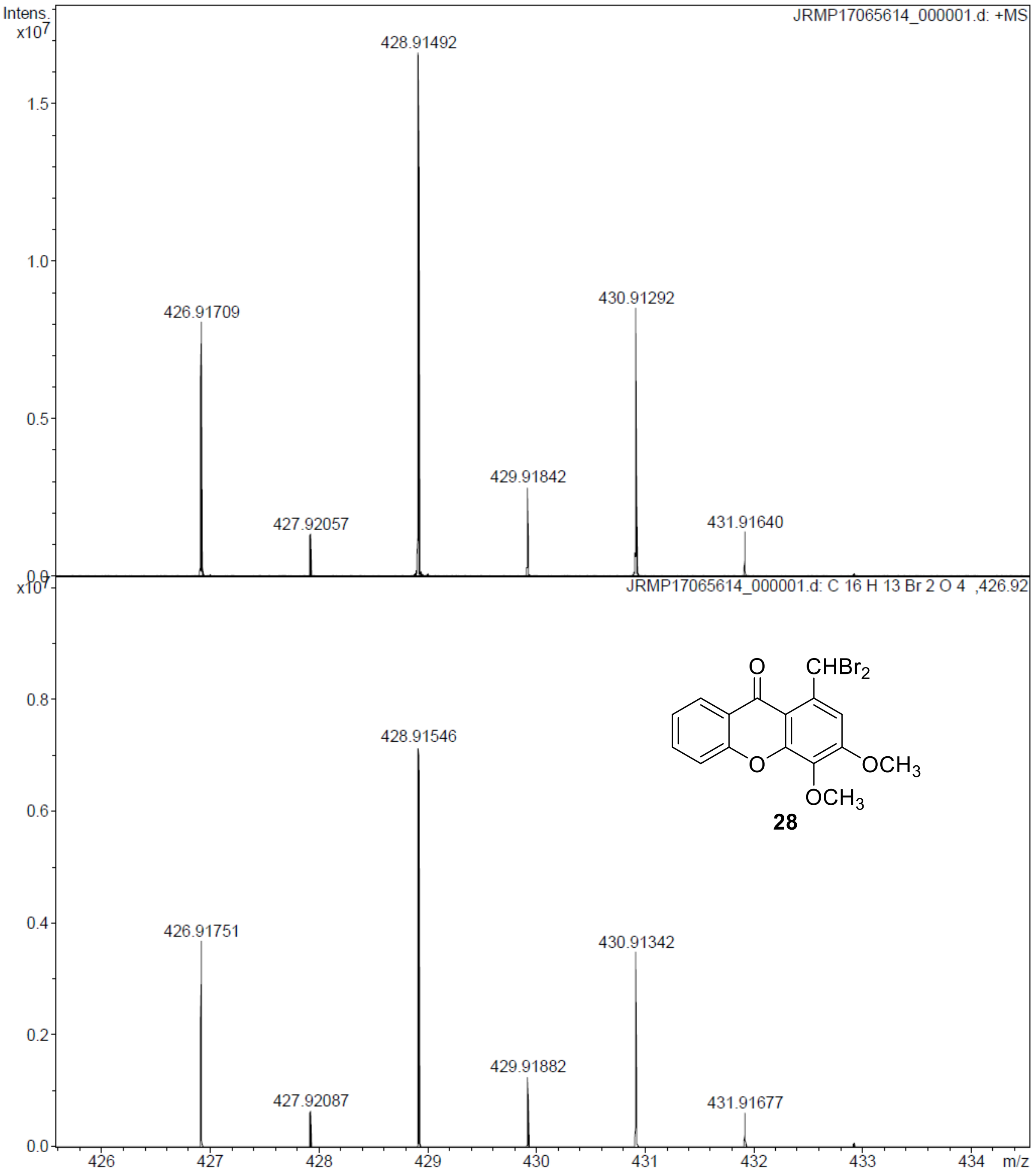

Figure S3. HRMS spectrum of 1-(dibromomethyl)-3,4-dimethoxy-9*H*-xanthen-9-one (**28**).

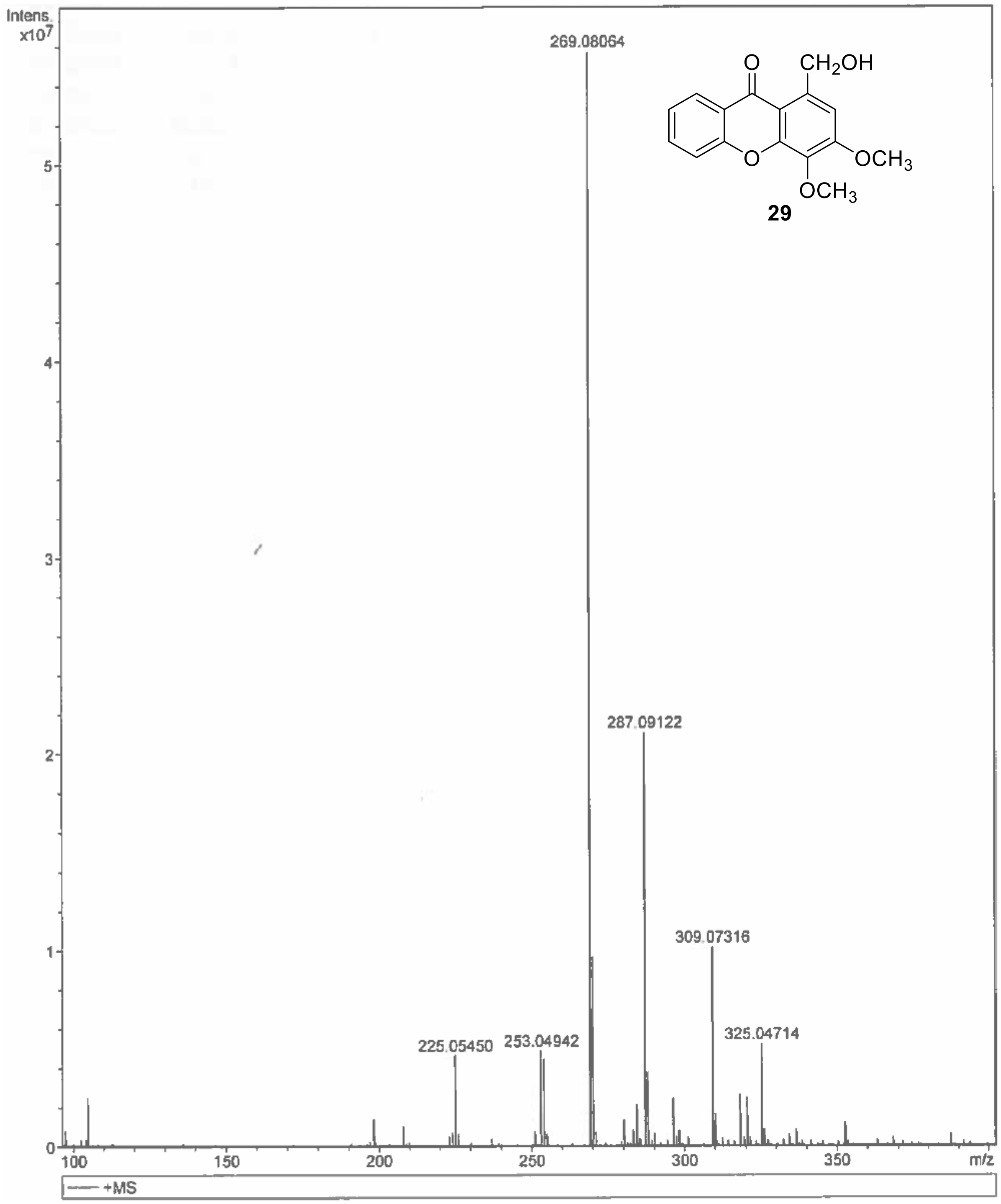

Intens.
x10^7
269.08064
287.09122
309.07316
325.04714
225.05450
253.04942
O
CH2OH
O
OCH3
OCH3
29
0
1
2
3
4
5
100
150
200
250
300
350
m/z
+MS

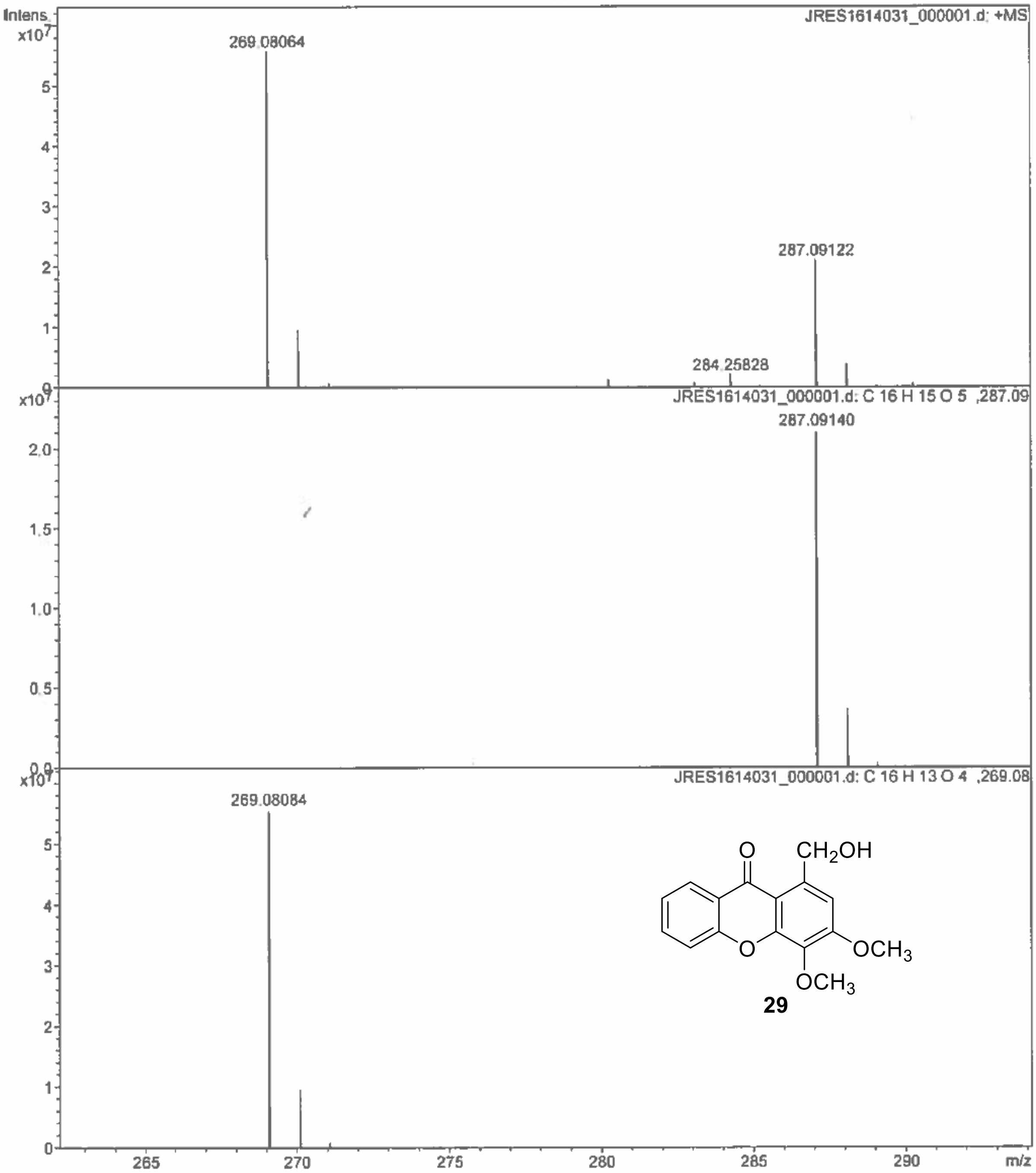

Figure S4. HRMS spectrum of 1-(hydroxymethyl)-3,4-dimethoxy-9*H*-xanthen-9-one (**29**).

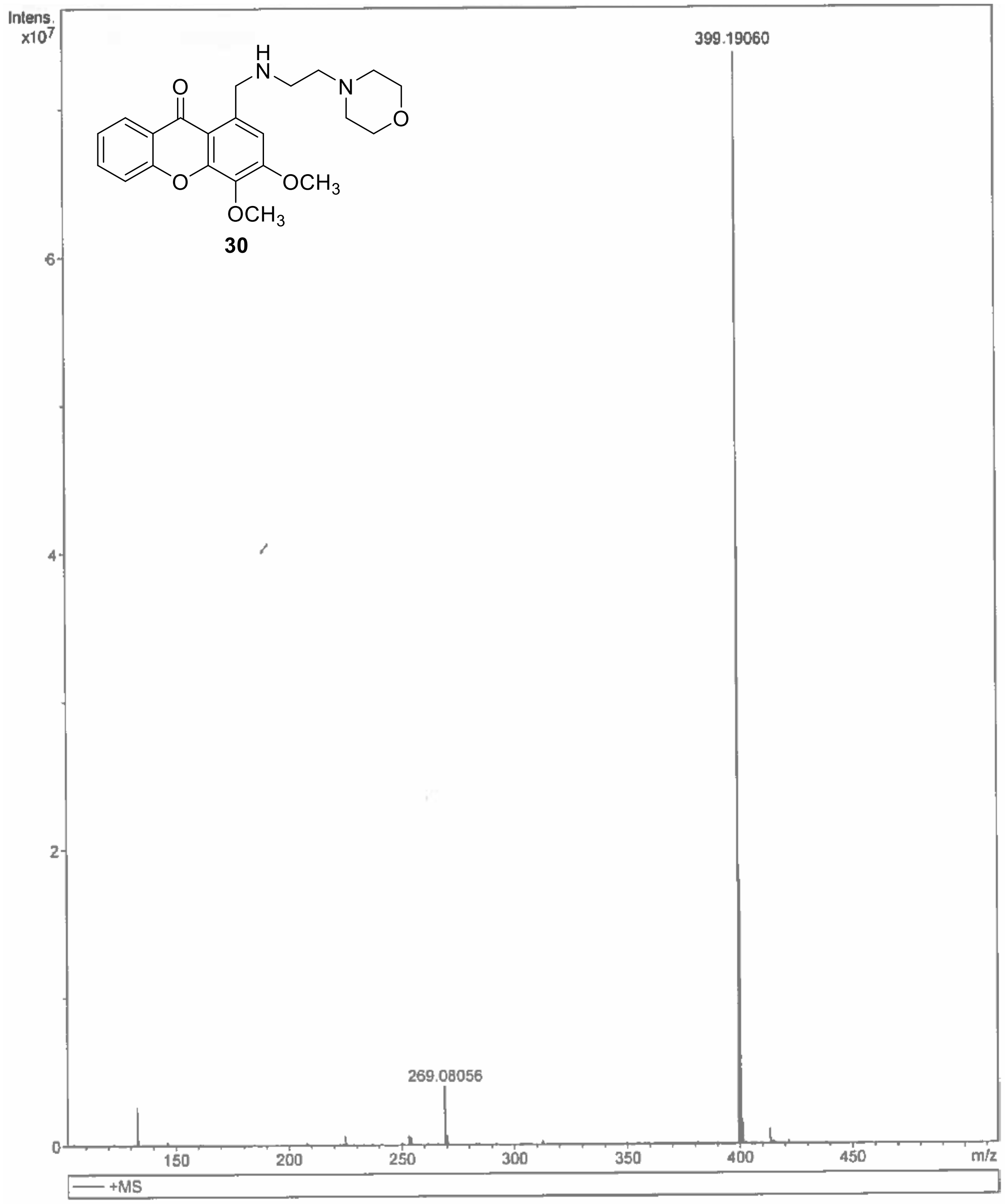

Intens.
x10^7
399.19060
269.08056
30
OCH3
OCH3
m/z
+MS

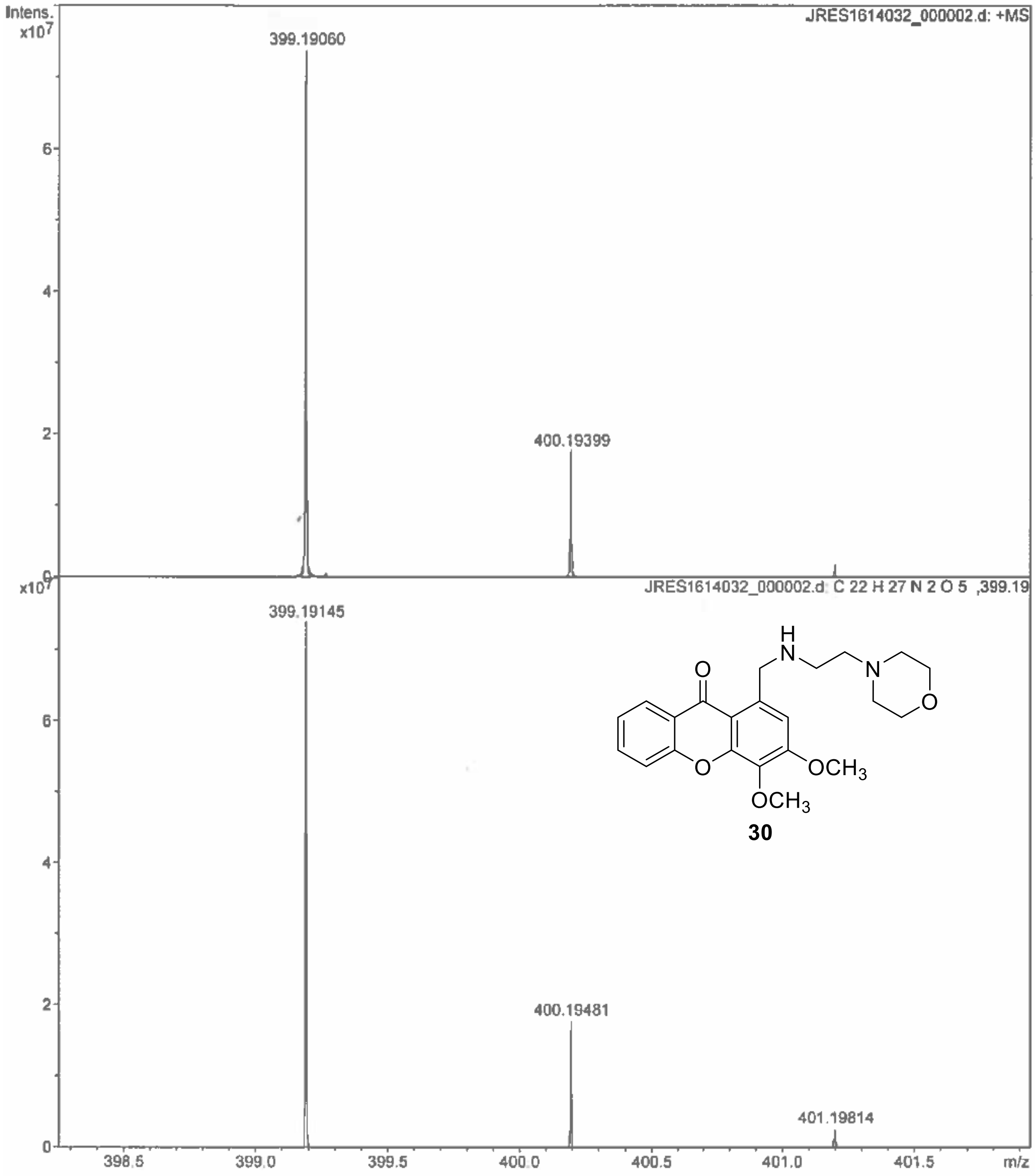

Figure S5. HRMS spectrum of 3,4-dimethoxy-1-(((2-morpholinoethyl)amino)methyl)-9*H*-xanthen-9-one (**30**).

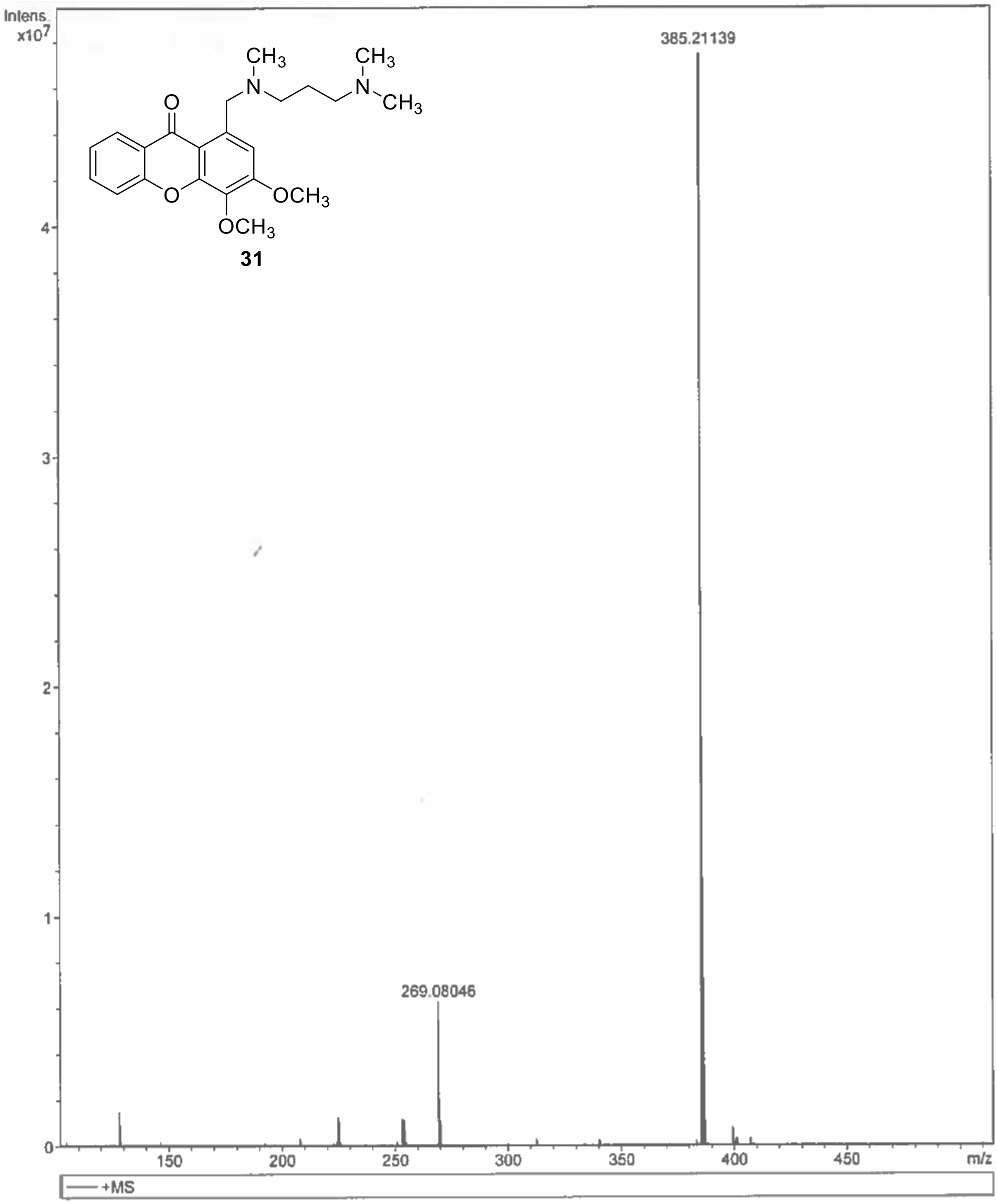
Intens.
x10^7
385.21139
269.08046
CH3
N
CH3
N
CH3
O
O
OCH3
OCH3
31
4
3
2
1
0
150
200
250
300
350
400
450
m/z
+MS

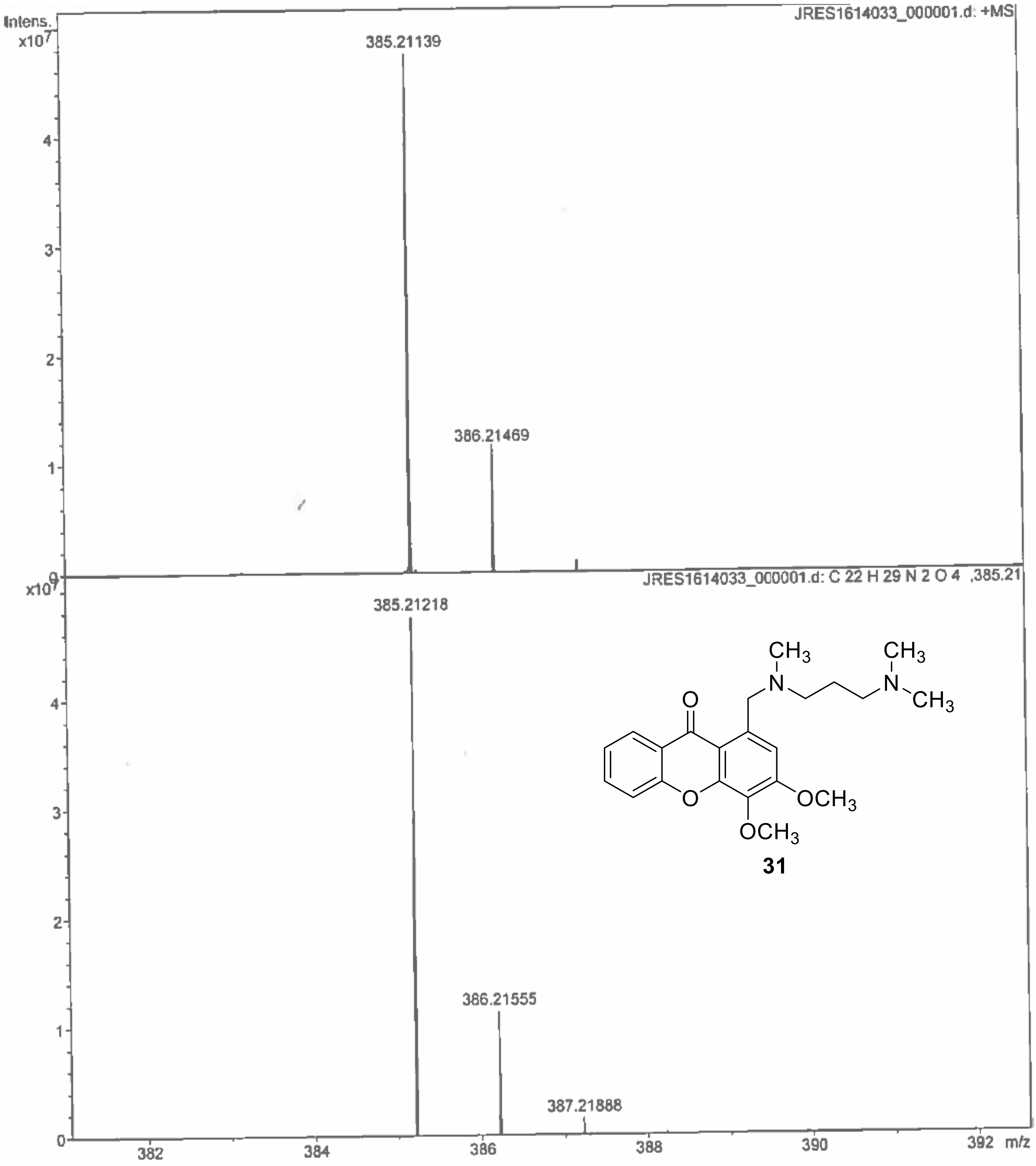

Figure S6. HRMS spectrum of 1-(((3-(dimethylamino)propyl)(methyl)amino)methyl)-3,4-dimethoxy-9*H*-xanthen-9-one (**31**).

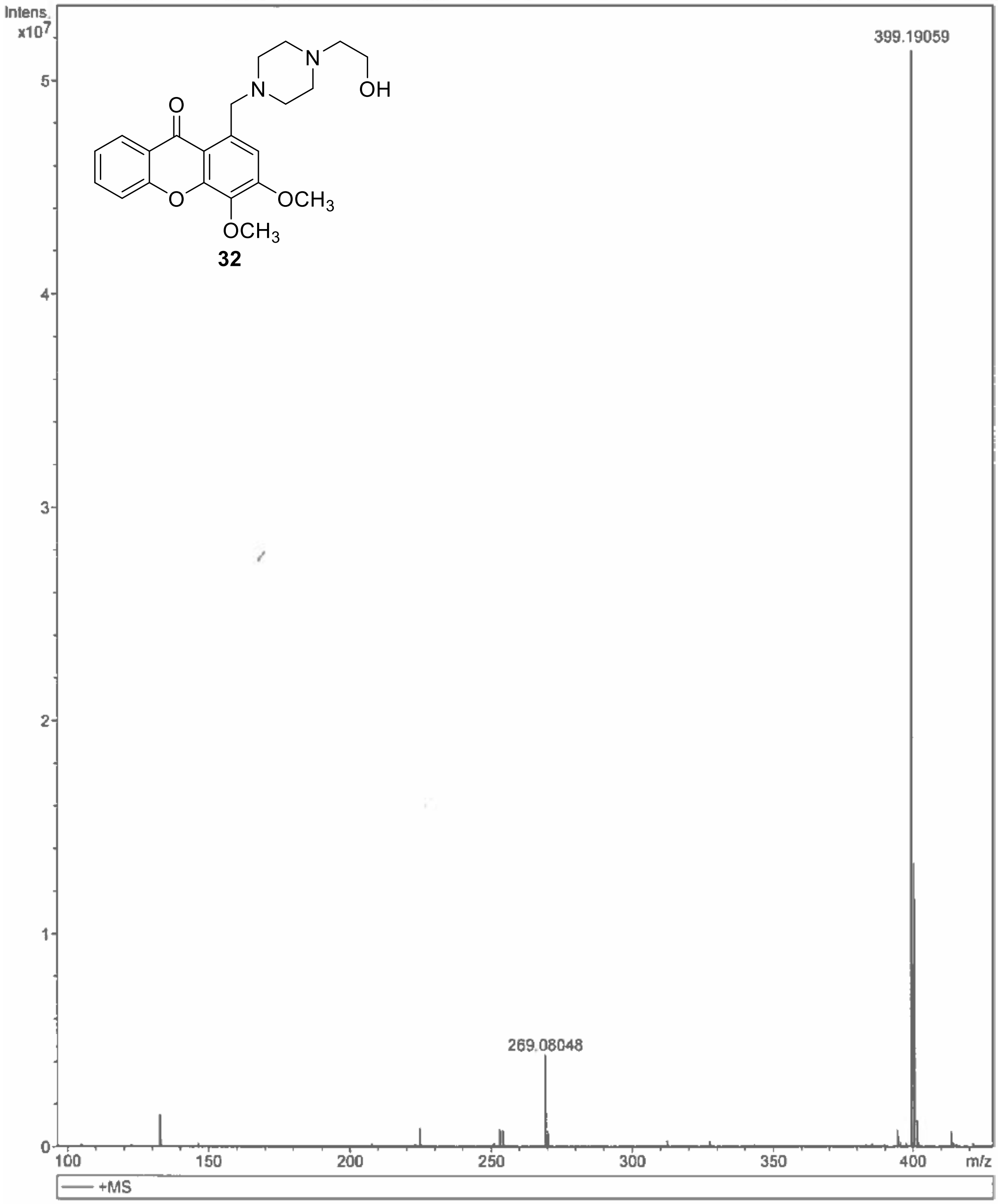
Intens.
x10^7
399.19059
269.08048
m/z
+MS
O
N
N
OH
O
OCH3
OCH3
32

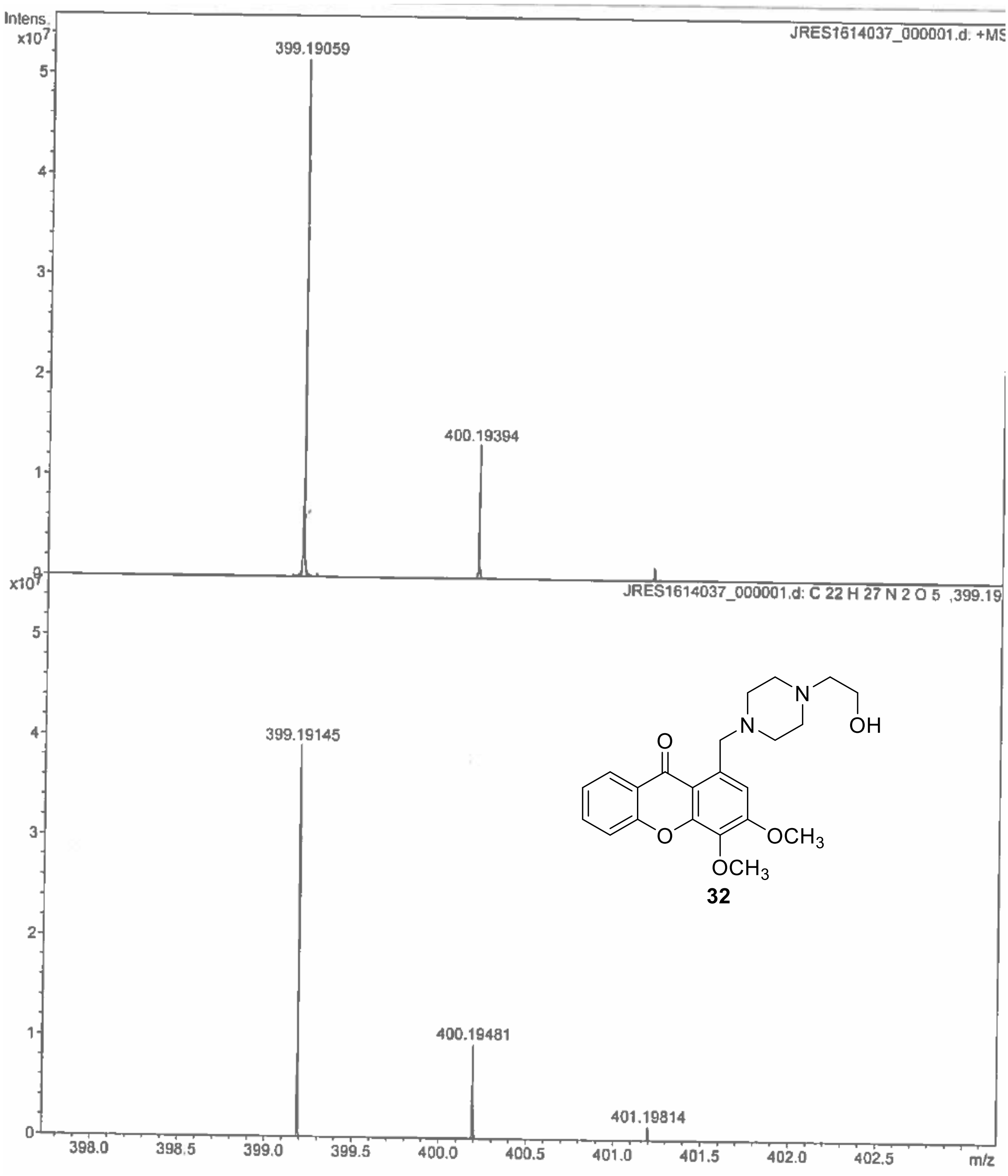

Figure S7. HRMS spectrum of 1-((4-(2-hydroxyethyl)piperazin-1-yl)methyl)-3,4-dimethoxy-9*H*-xanthen-9-one (**32**).

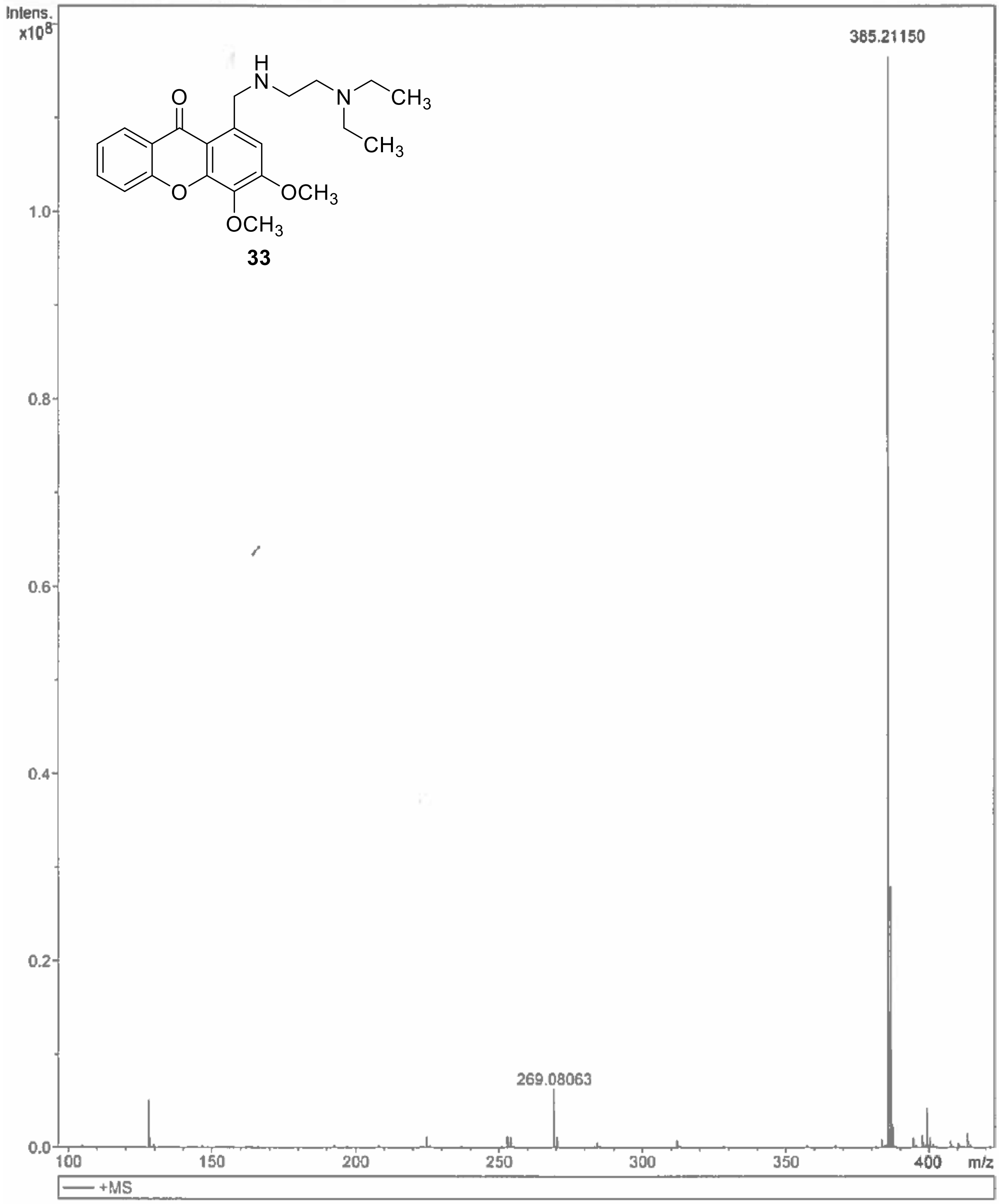
Intens.
x10^8
385.21150
269.08063
H
N
O
CH3
CH3
OCH3
OCH3
33
1.0
0.8
0.6
0.4
0.2
0.0
100
150
200
250
300
350
400
m/z
+MS

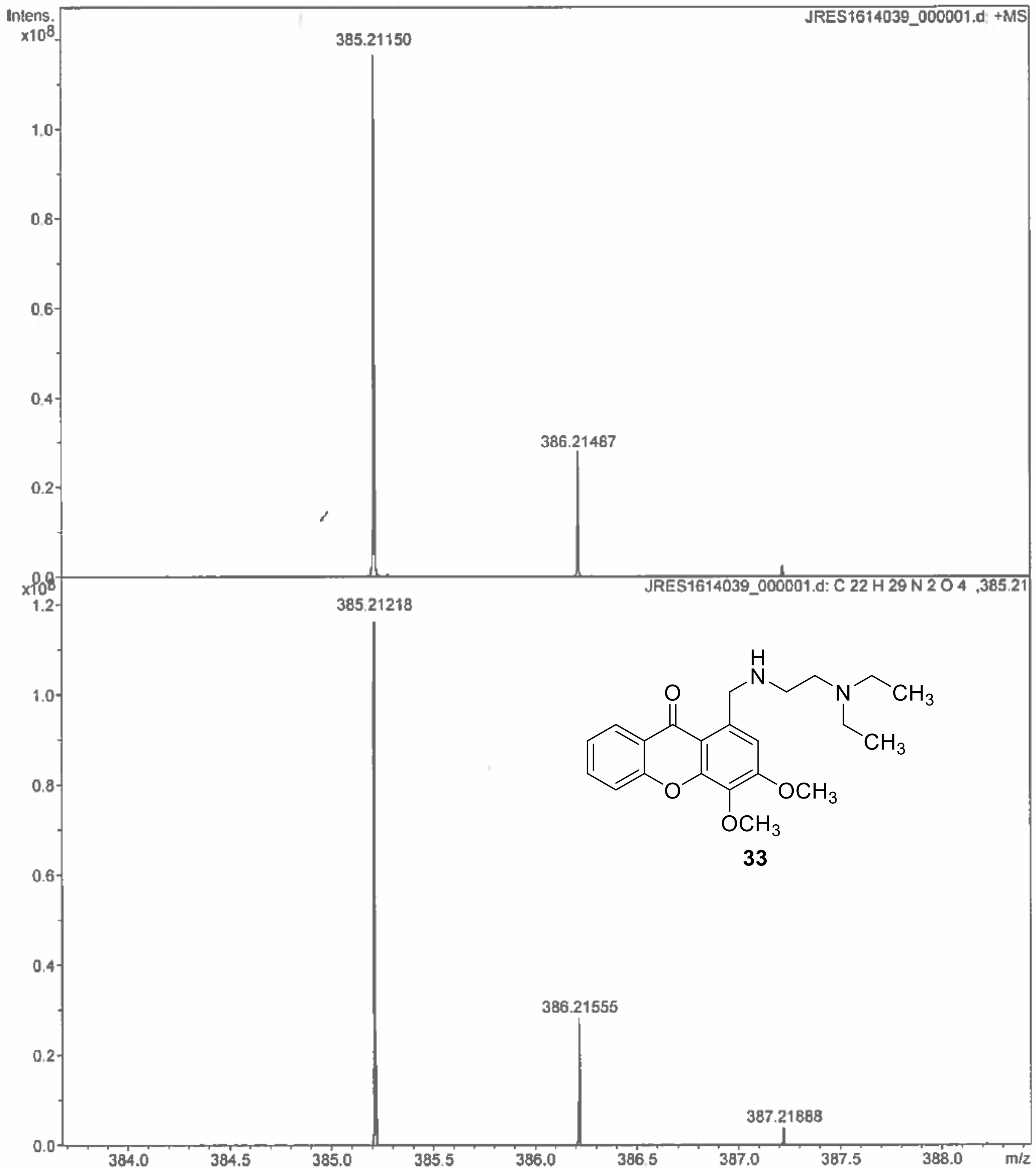

Figure S8. HRMS spectrum of 1-(((2-(diethylamino)ethyl)amino)methyl)-3,4-dimethoxy-9*H*-xanthen-9-one (**33**).

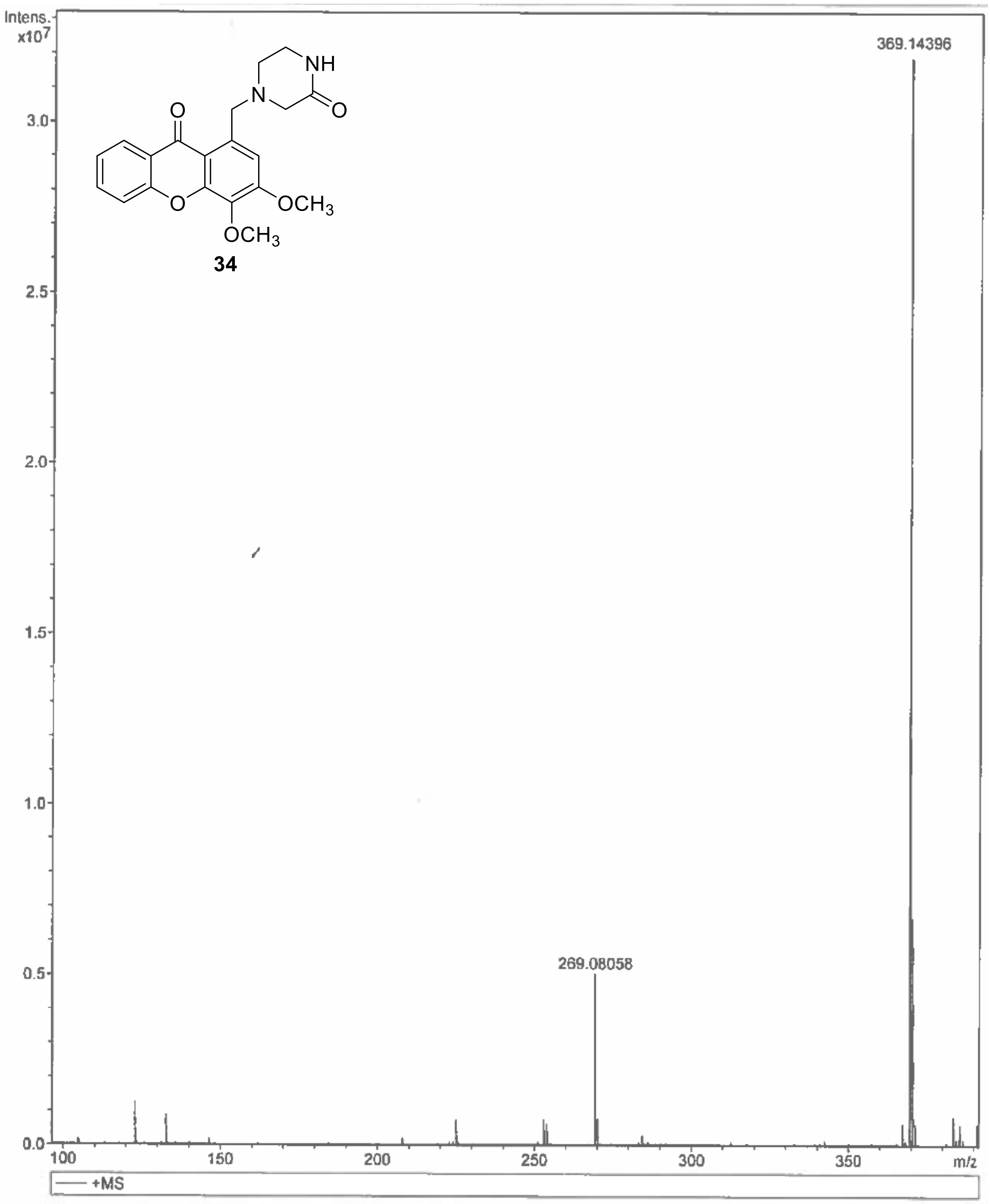

Intens.
x10^7
3.0
2.5
2.0
1.5
1.0
0.5
0.0
369.14396
269.08058
100
150
200
250
300
350
m/z
+MS
NH
N
O
O
O
OCH3
OCH3
34

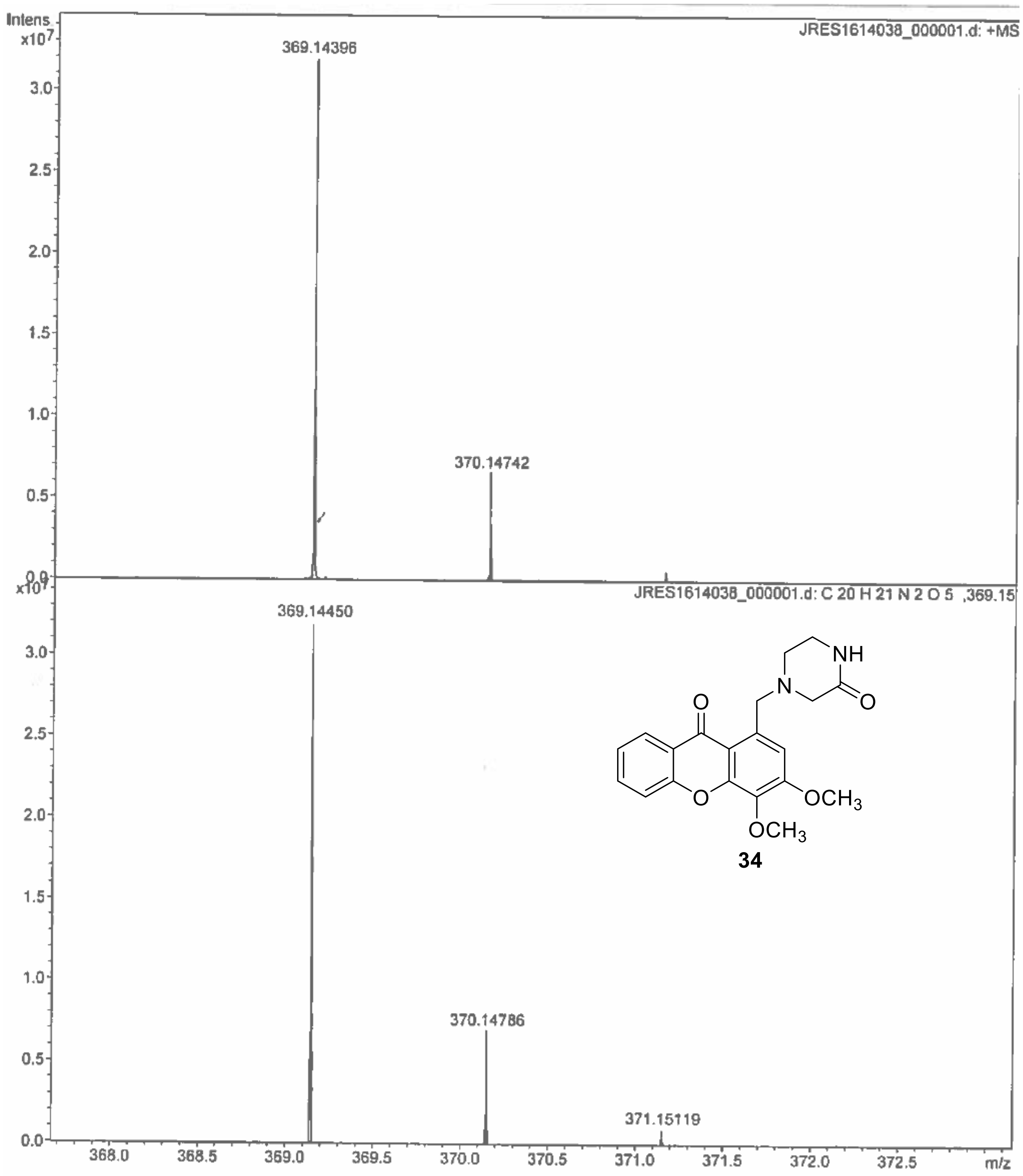

Figure S9. HRMS spectrum of 4-((3,4-dimethoxy-9-oxo-9*H*-xanthen-1-yl)methyl)piperazin-2-one (**34**).

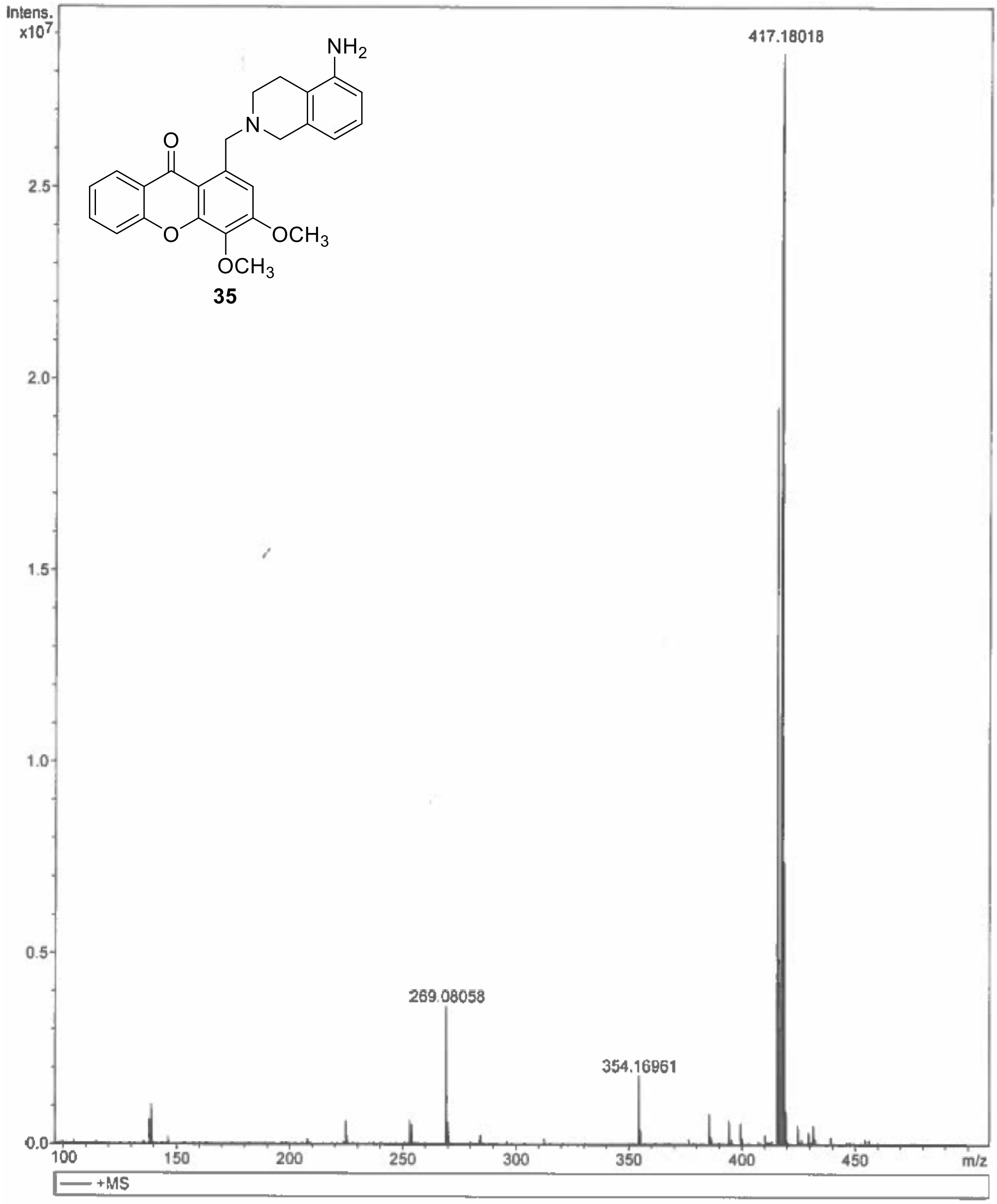
Intens.
x10^7
2.5
2.0
1.5
1.0
0.5
0.0
100
150
200
250
300
350
400
450
m/z
417.18018
269.08058
354.16961
+MS
NH2
N
O
O
OCH3
OCH3
35

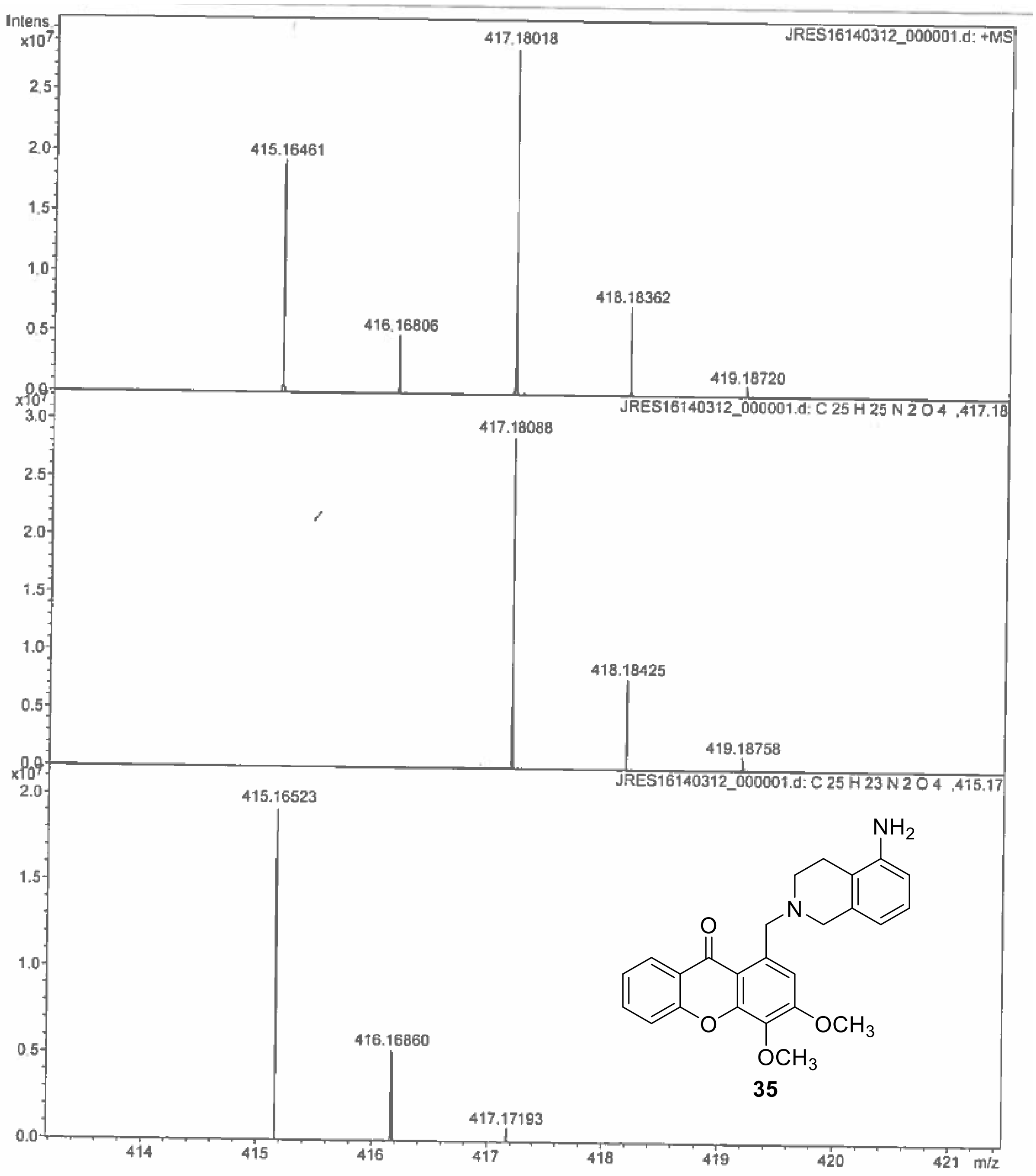

Figure S10. HRMS spectrum of 1-((5-amino-3,4-dihydroisoquinolin-2(1*H*)-yl)methyl)-3,4-dimethoxy-9*H*-xanthen-9-one (**35**).

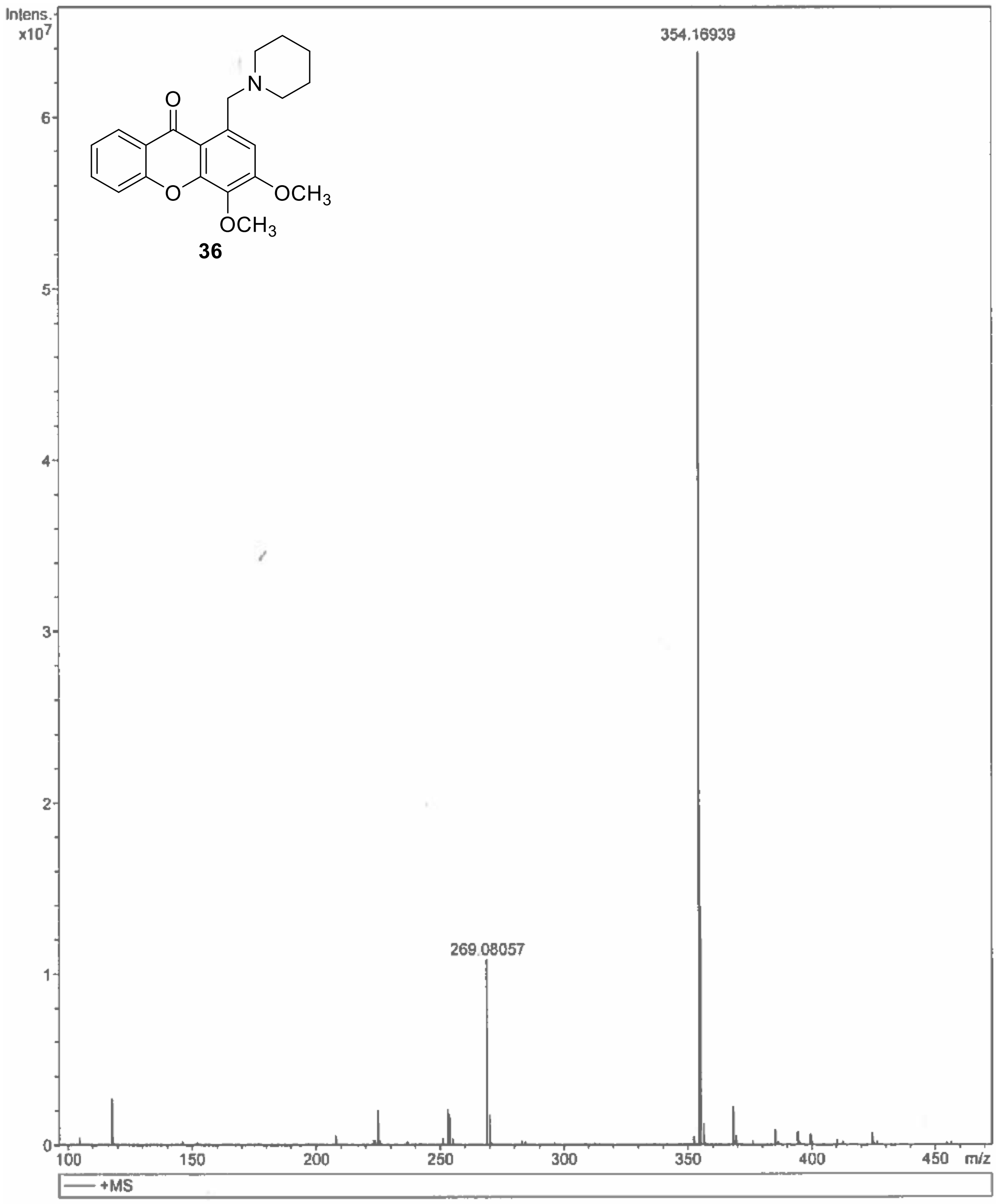
Intens.
x10^7
354.16939
269.08057
OCH3
OCH3
36
m/z
+MS

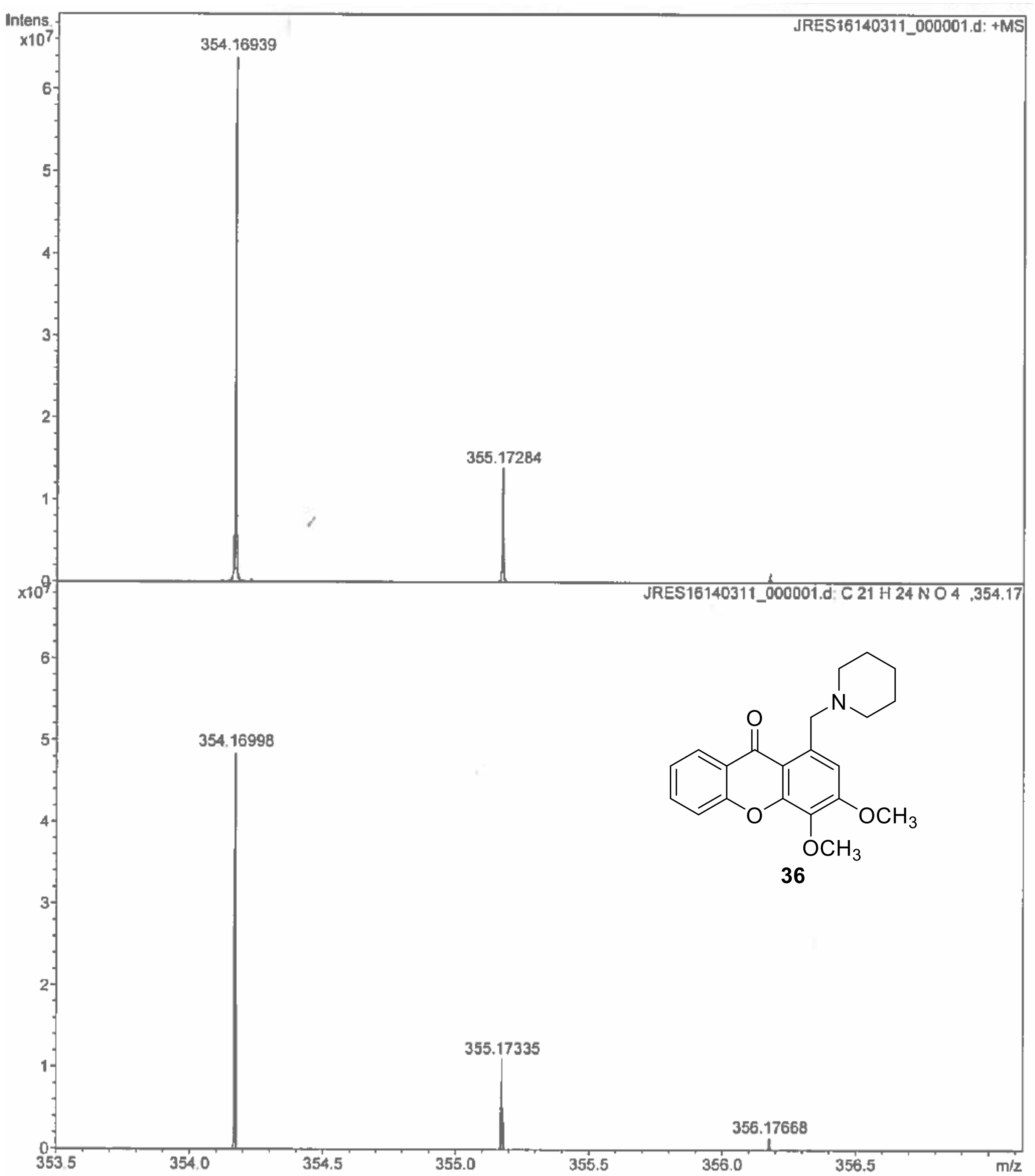

Figure S11. HRMS spectrum of 3,4-dimethoxy-1-(piperidin-1-ylmethyl)-9*H*-xanthen-9-one (**36**).

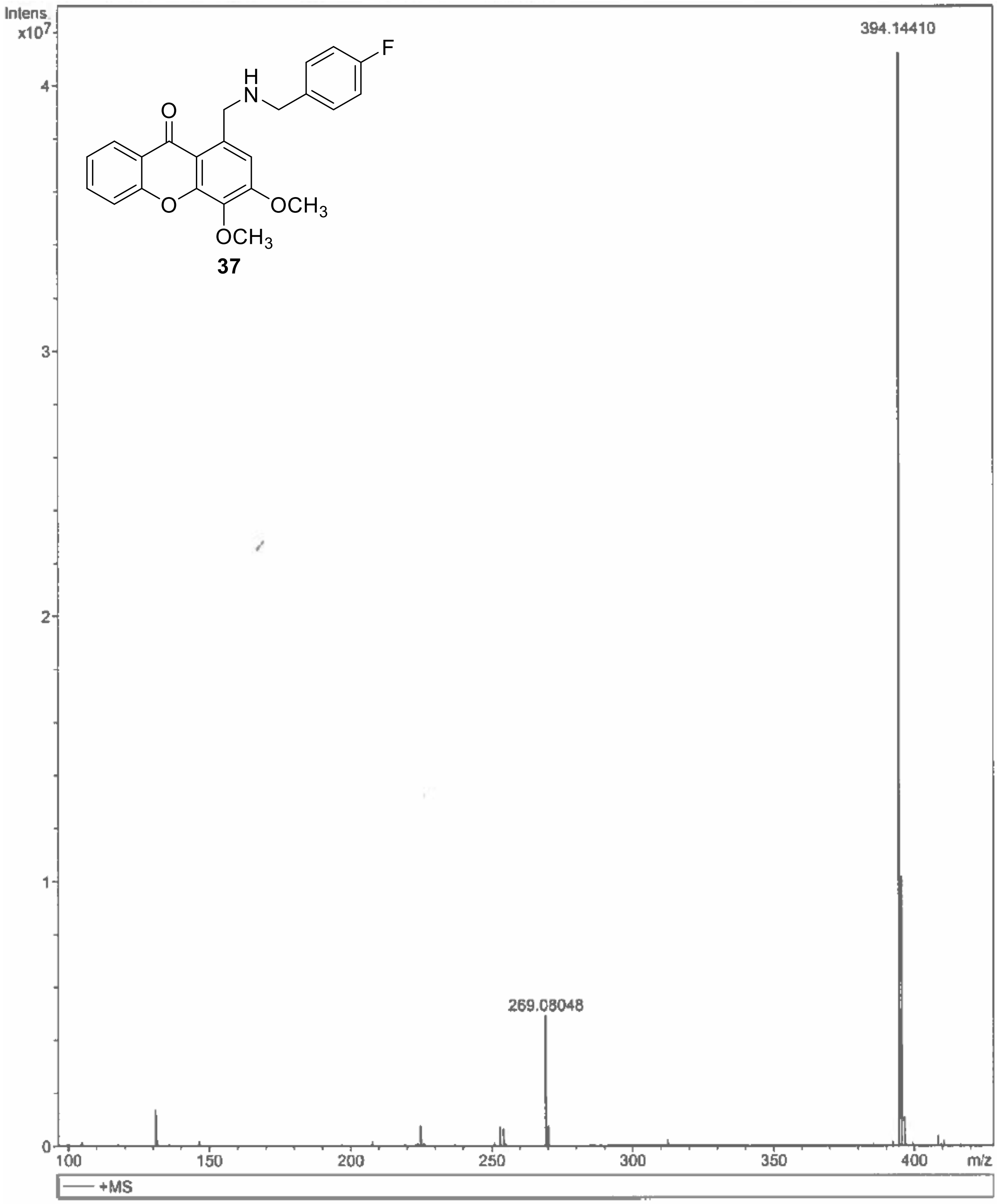
Intens.
x10^7
4
3
2
1
0
394.14410
269.08048
100
150
200
250
300
350
400
m/z
+MS
H
N
F
O
O
OCH3
OCH3
37

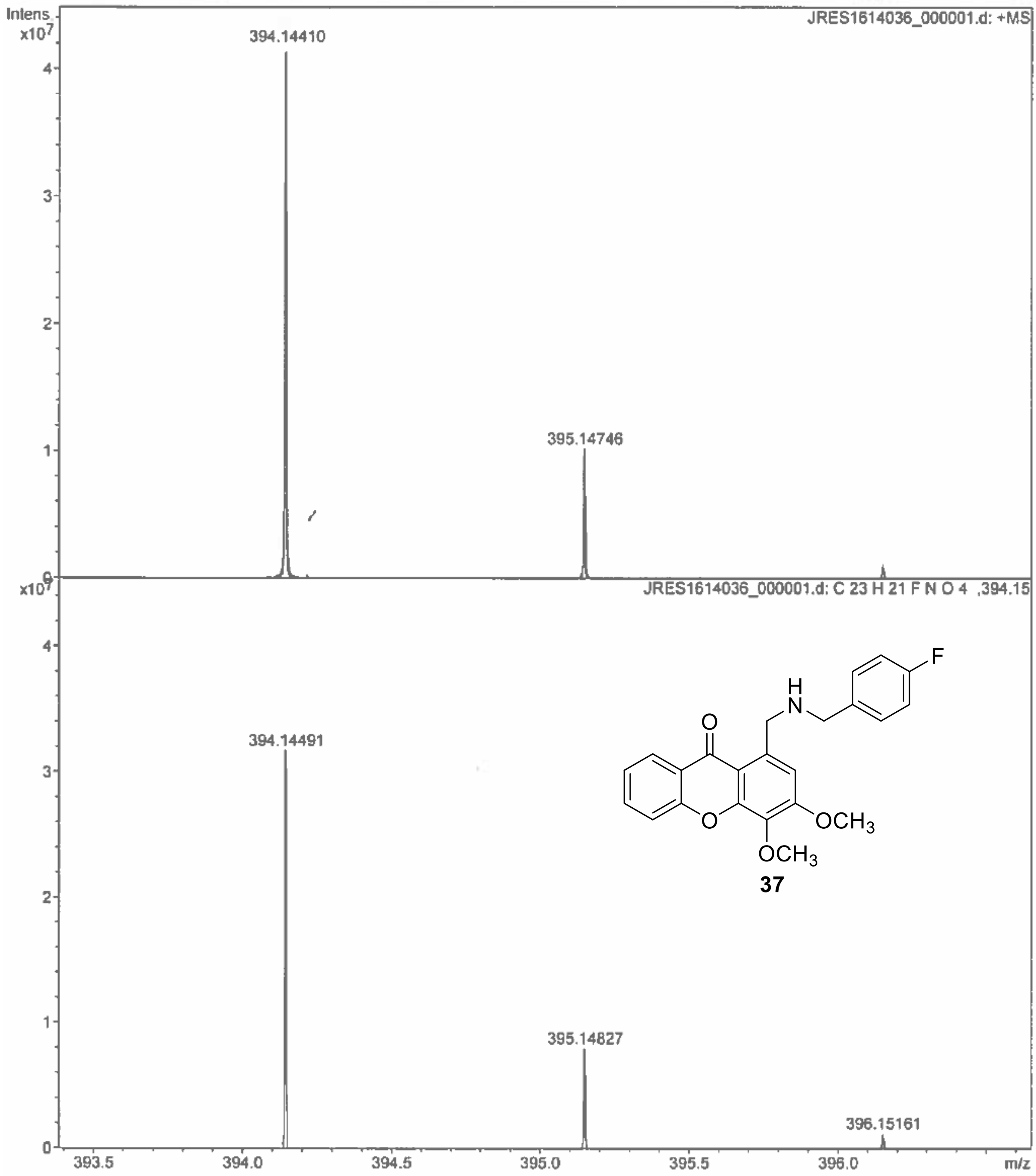

Figure S12. HRMS spectrum of 1-(((4-fluorobenzyl)amino)methyl)-3,4-dimethoxy-9*H*-xanthen-9-one (**37**).

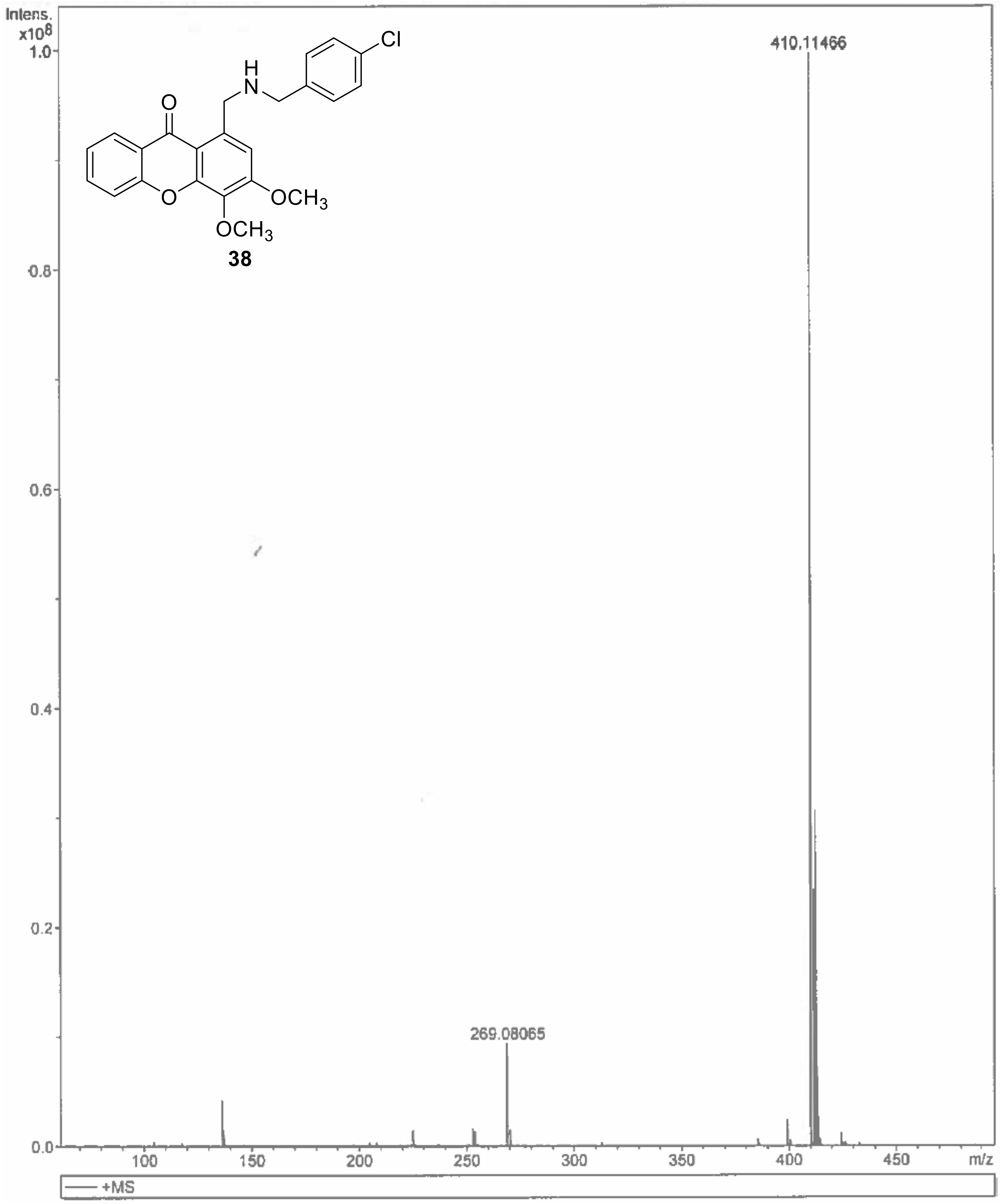

Intens.
x10^8
1.0
0.8
0.6
0.4
0.2
0.0
H
N
Cl
O
O
OCH3
OCH3
38
410.11466
269.08065
100
150
200
250
300
350
400
450
m/z
+MS

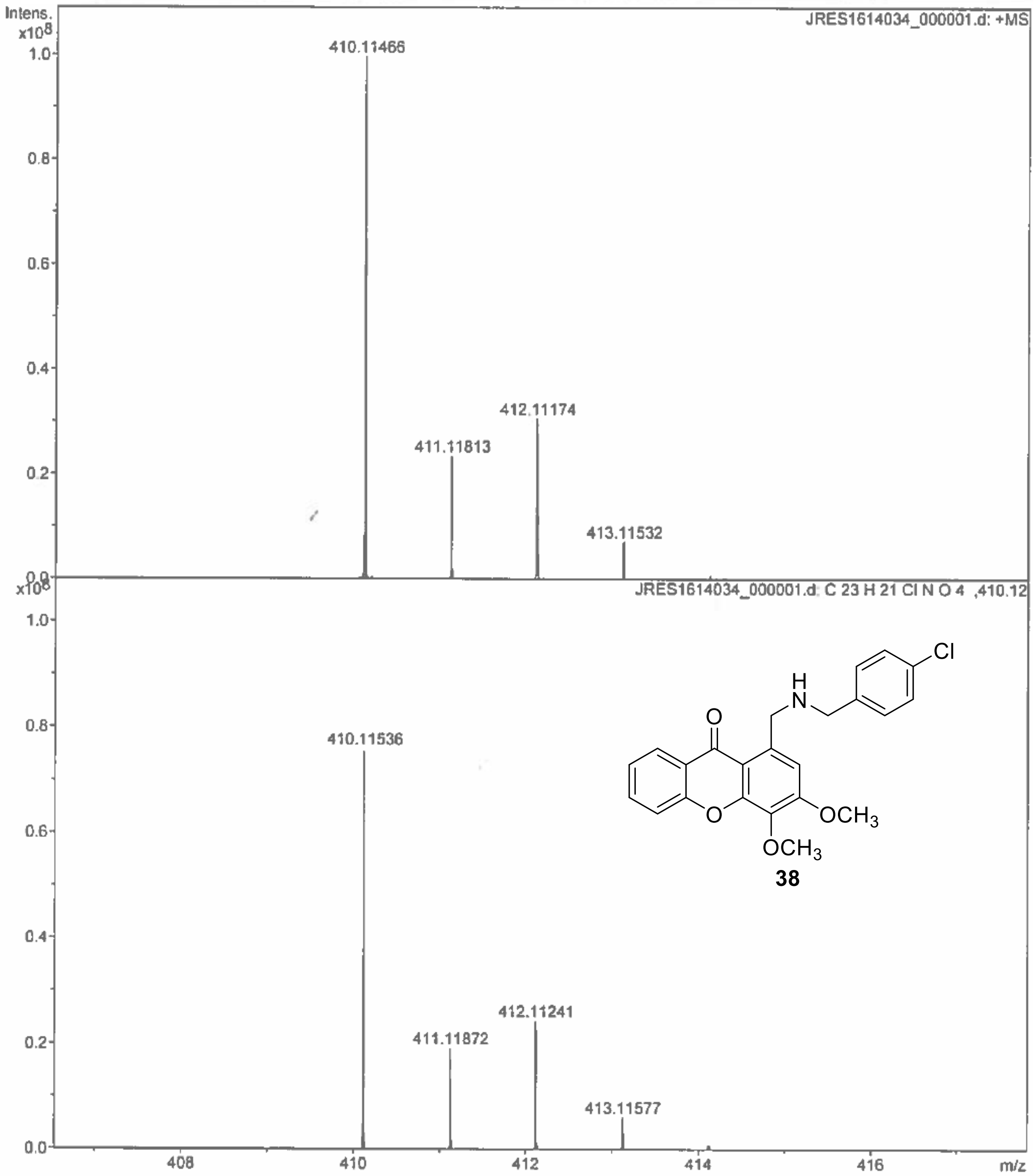

Figure S13. HRMS spectrum of 1-(((4-chlorobenzyl)amino)methyl)-3,4-dimethoxy-9*H*-xanthen-9-one (**38**).

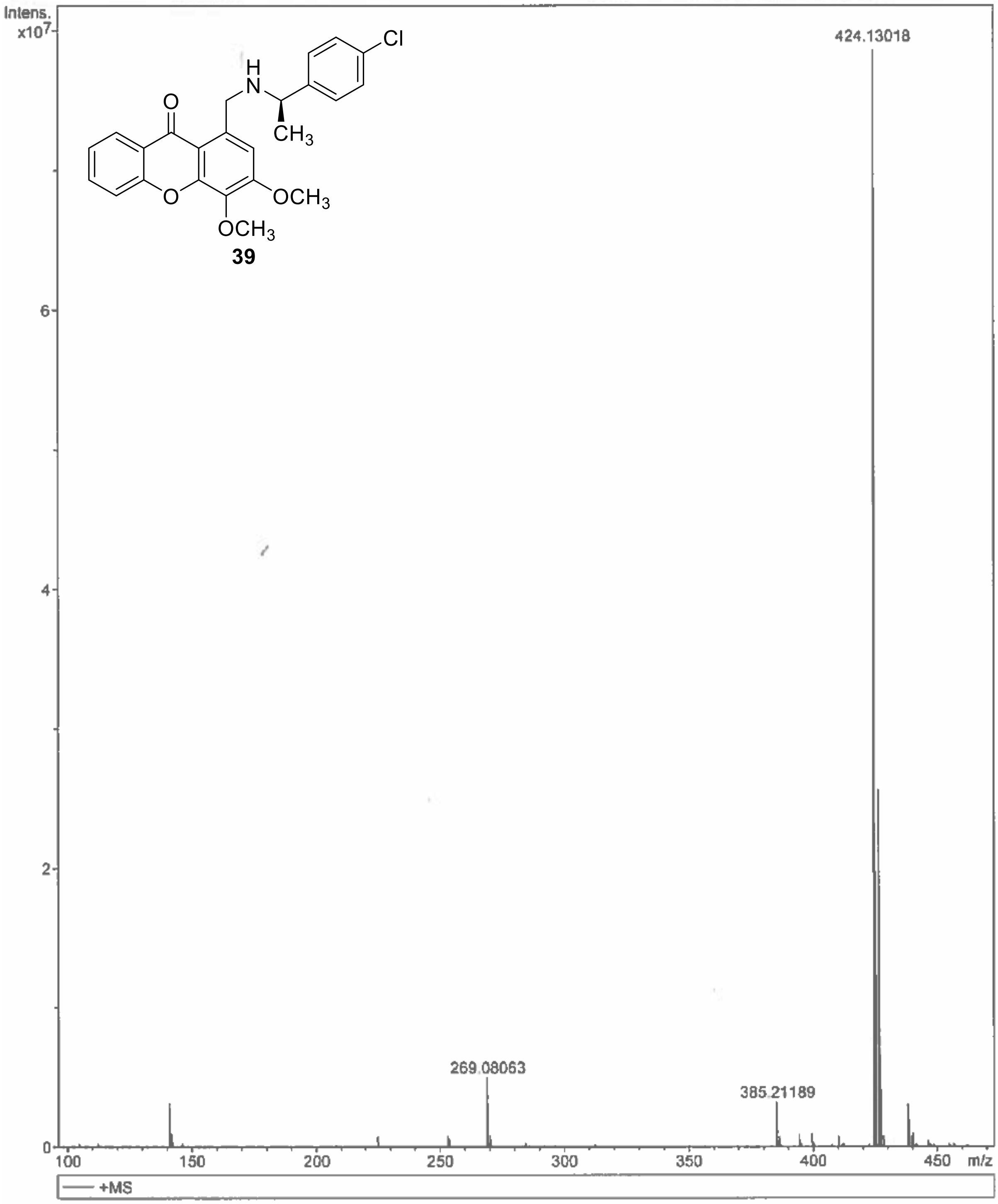

Intens.
x10^7
6
4
2
0
100
150
200
250
300
350
400
450
m/z
424.13018
269.08063
385.21189
+MS
O
H
N
Cl
CH3
O
OCH3
OCH3
39

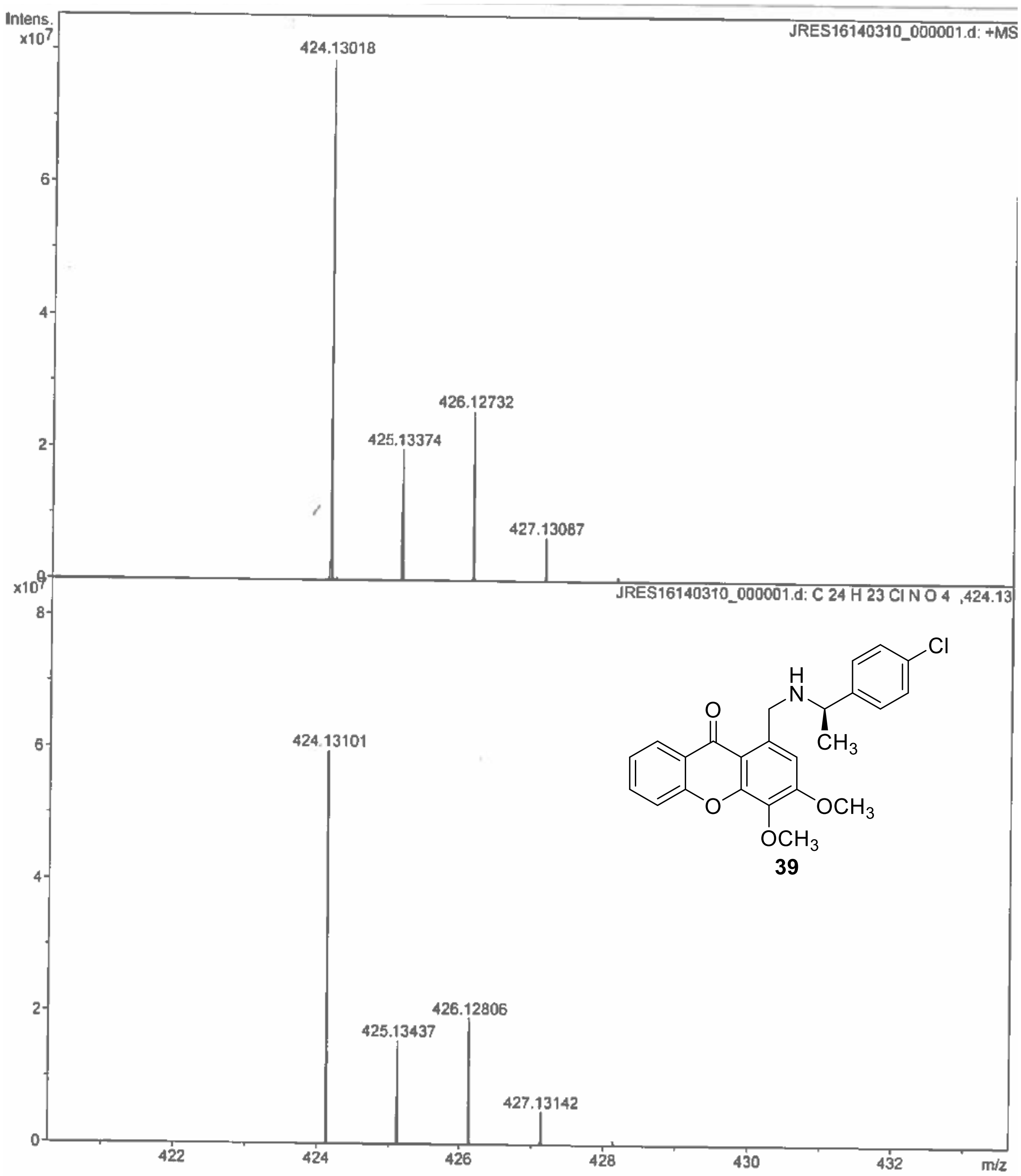

Figure S14. HRMS spectrum of (*R*)-1-(((1-(4-chlorophenyl)ethyl)amino)methyl)-3,4-dimethoxy-9*H*-xanthen-9-one (**39**).

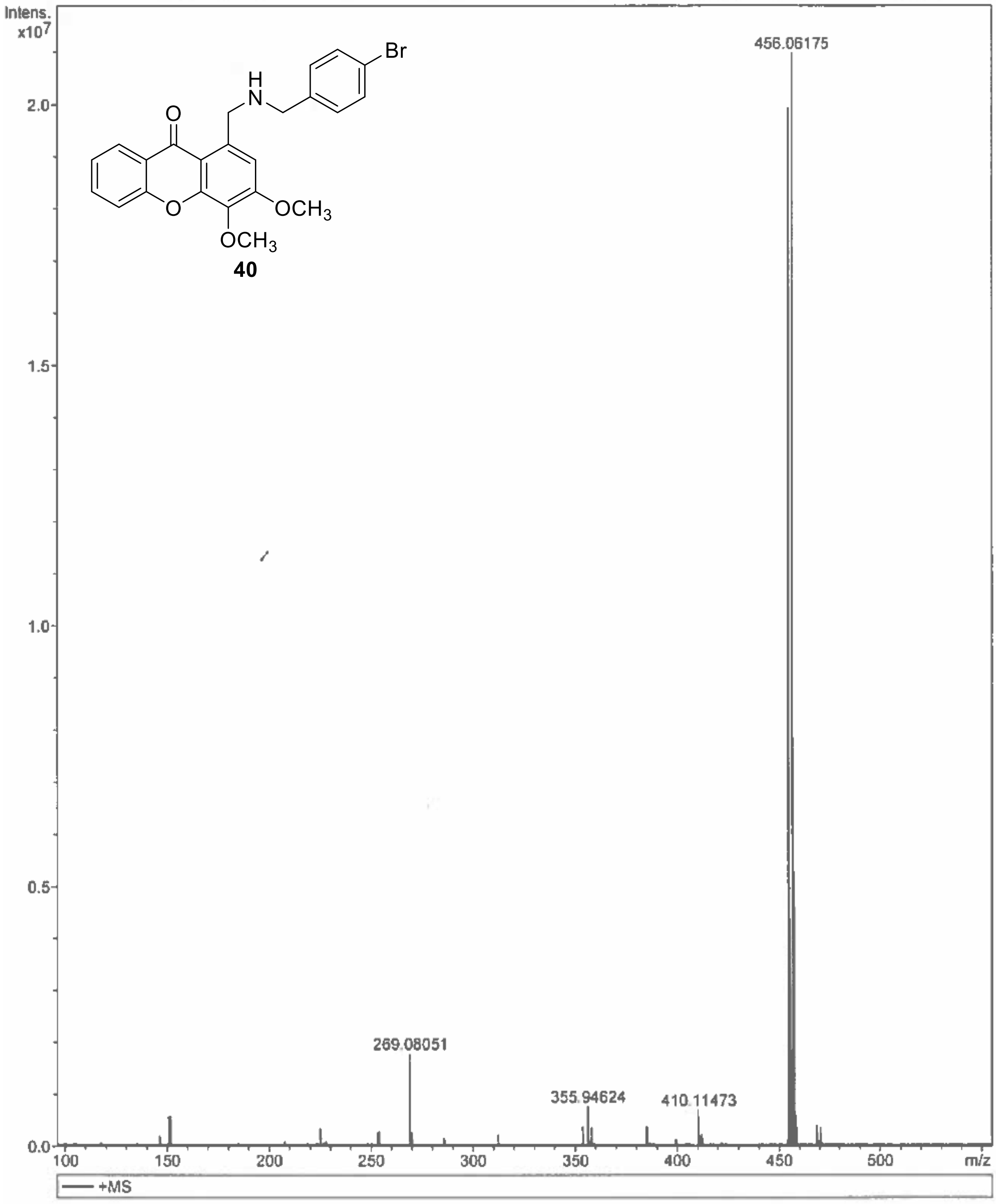

Intens.
x10^7
2.0
1.5
1.0
0.5
0.0
Br
H
N
O
O
OCH3
OCH3
40
456.06175
269.08051
355.94624
410.11473
100
150
200
250
300
350
400
450
500
m/z
+MS

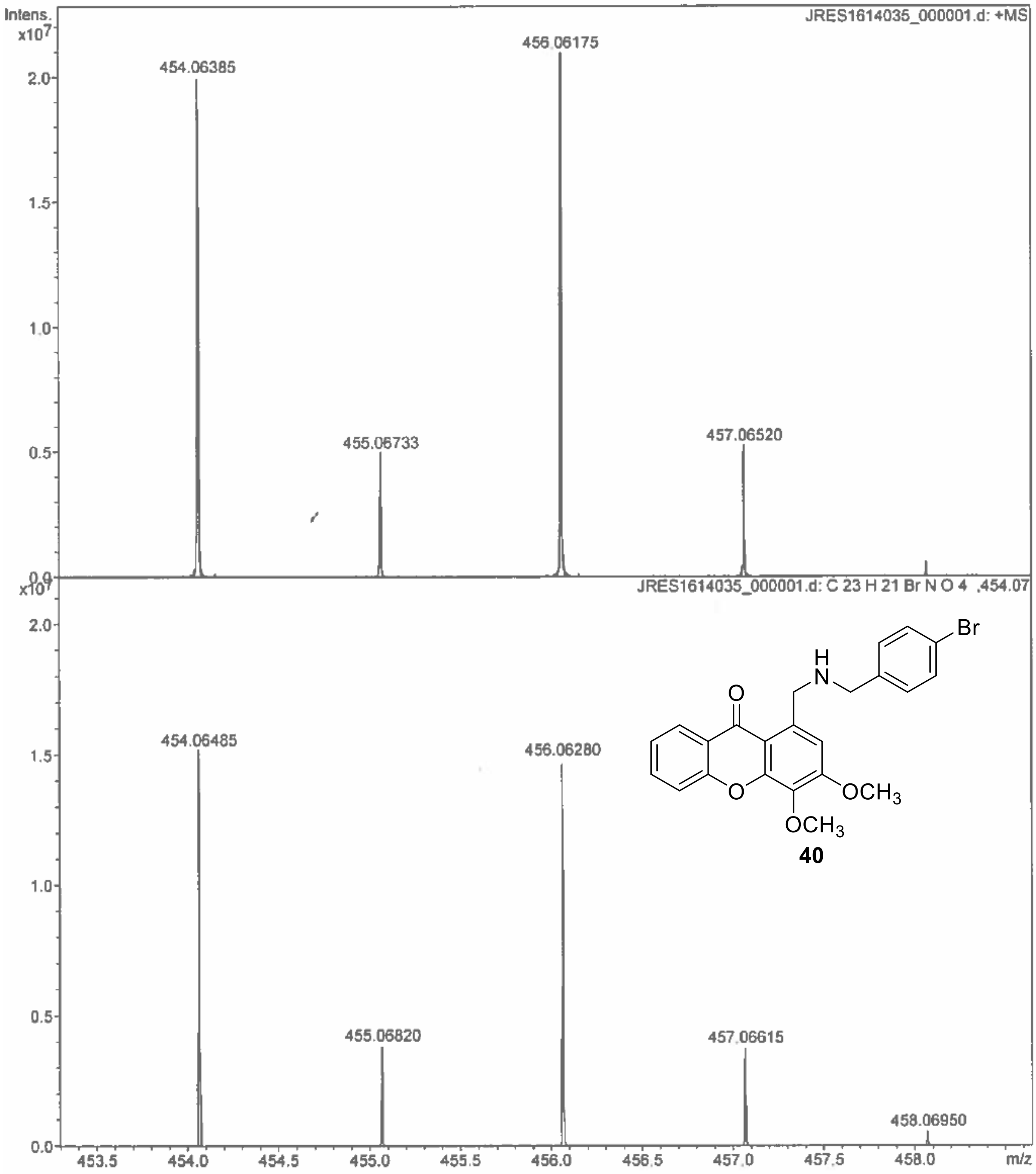

Figure S15. HRMS spectrum of 1-(((4-bromobenzyl)amino)methyl)-3,4-dimethoxy-9*H*-xanthen-9-one (**40**).

2. ^{1}H- and ^{13}C-NMR spectra of the described xanthone derivatives

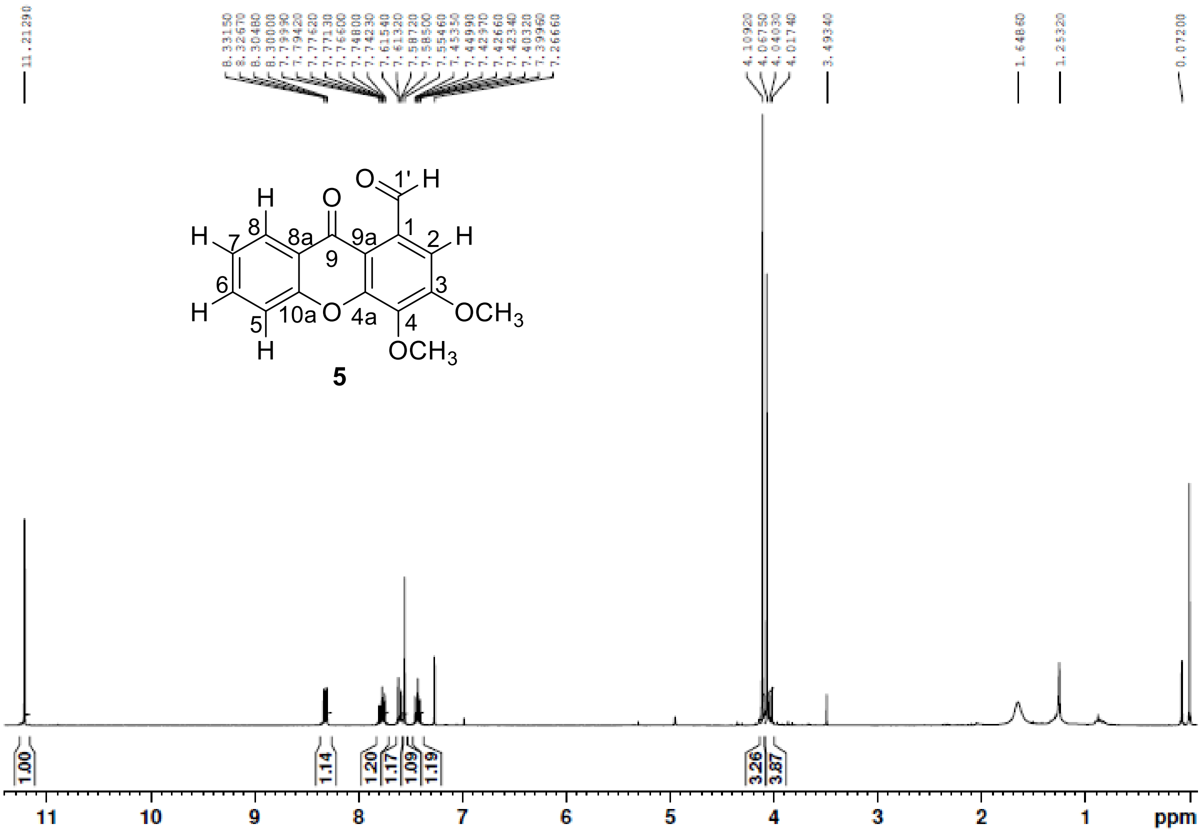

Figure S16. ^{1}H-NMR spectrum of 3,4-dimethoxy-9-oxo-9*H*-xanthene-1-carbaldehyde (**5**).

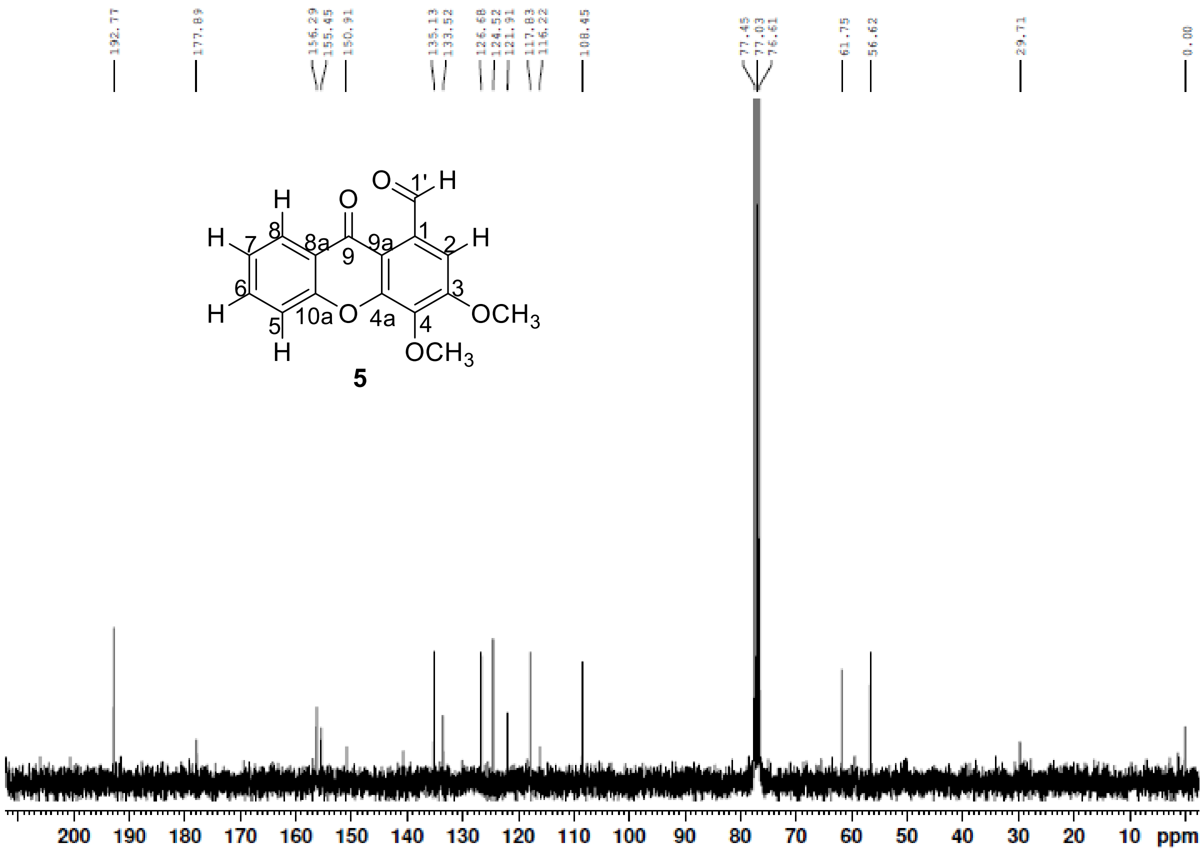

Figure S17. ^{13}C-NMR spectrum of 3,4-dimethoxy-9-oxo-9*H*-xanthene-1-carbaldehyde (**5**).

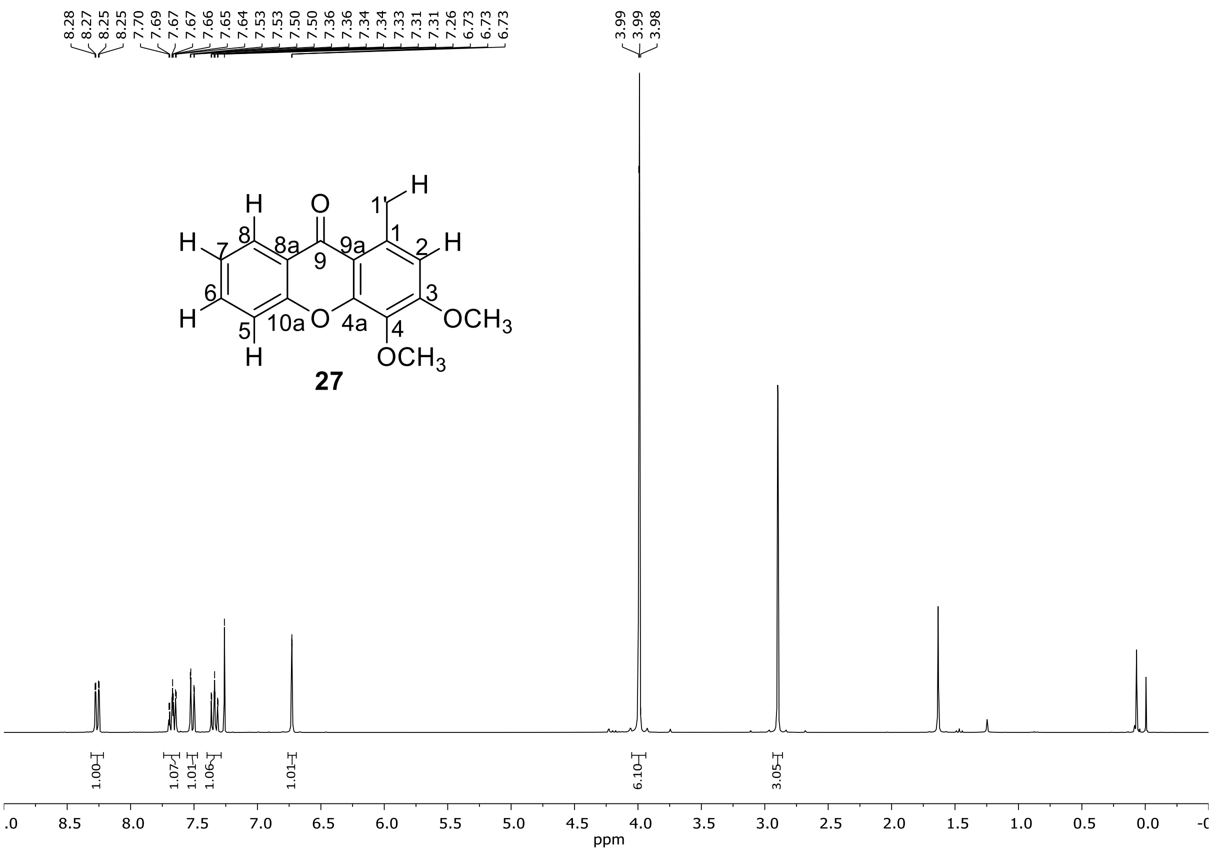

Figure S18. ^{1}H-NMR spectrum of 3,4-dimethoxy-1-methyl-9*H*-xanthen-9-one (**27**).

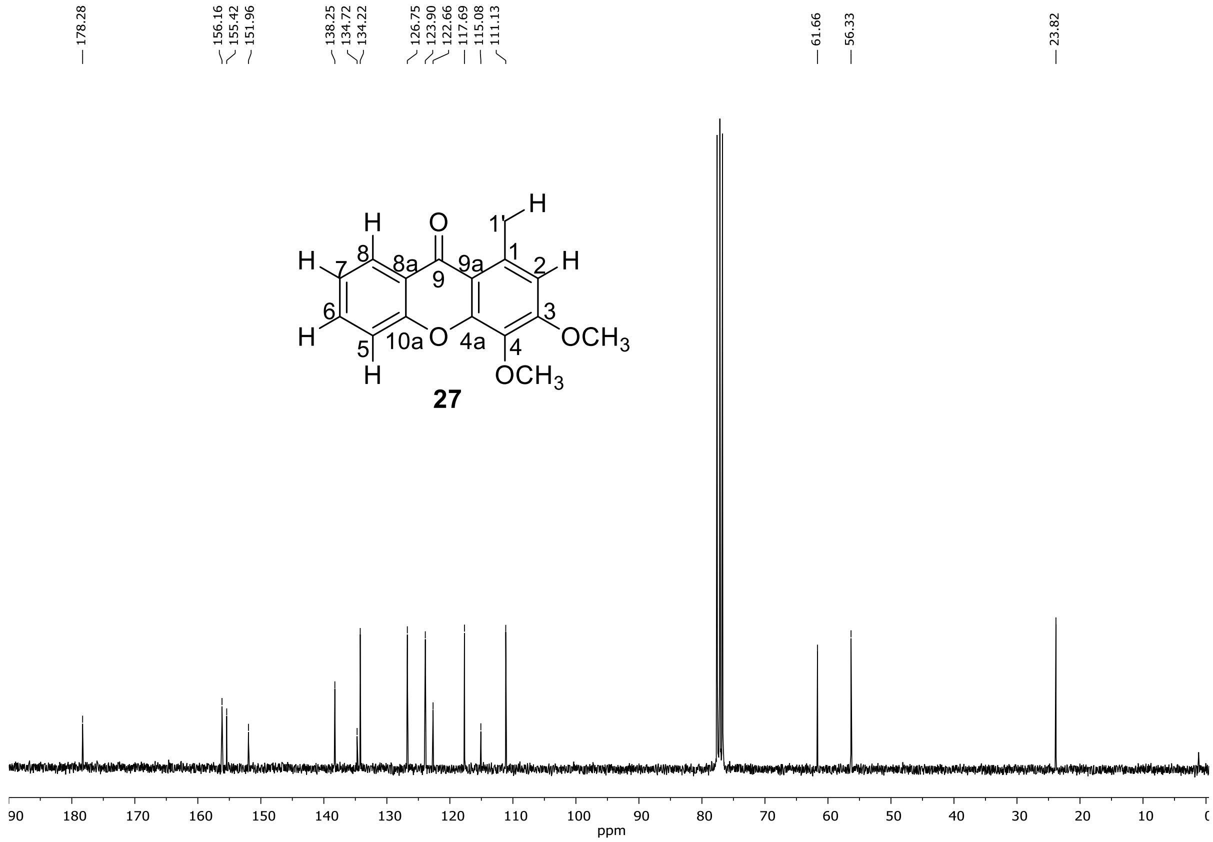

Figure S19. ^{13}C-NMR spectrum of 3,4-dimethoxy-1-methyl-9*H*-xanthen-9-one (**27**).

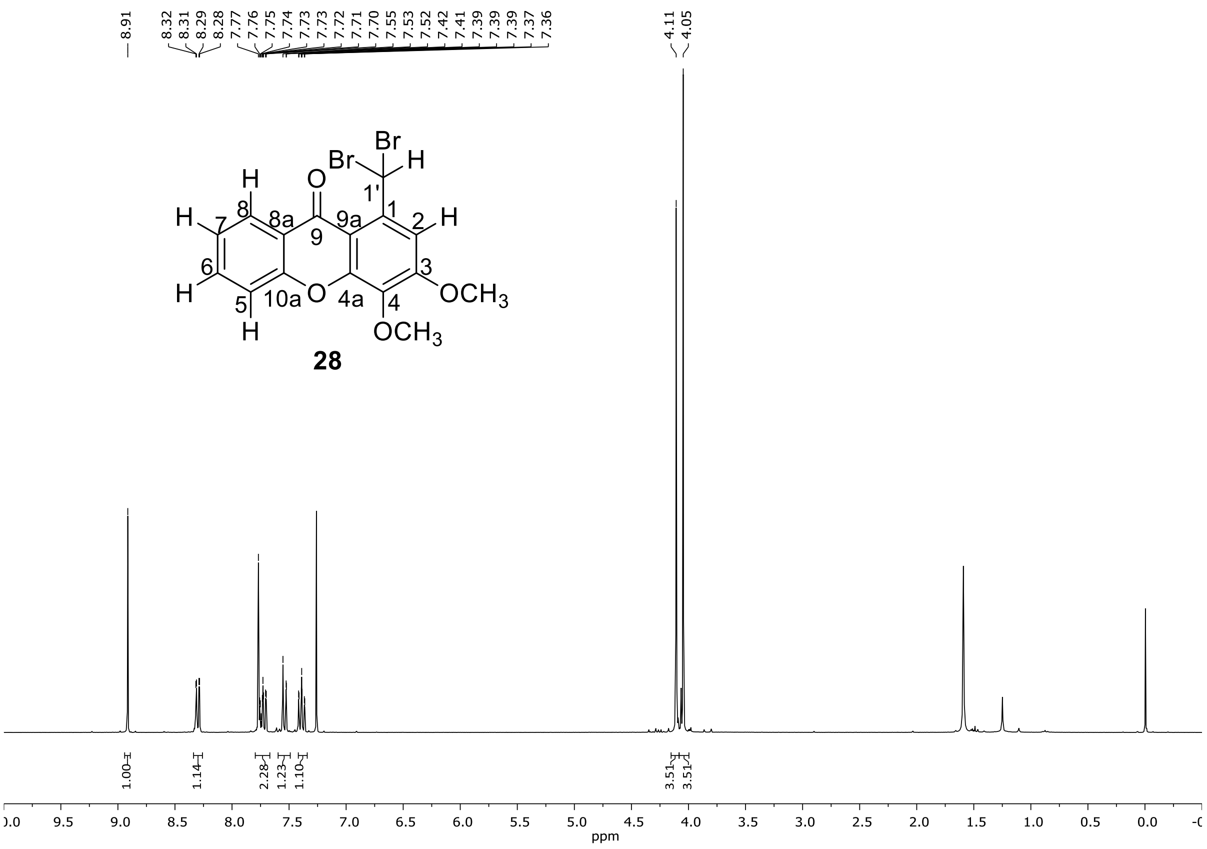

Figure S20. ^{1}H-NMR spectrum of 1-(dibromomethyl)-3,4-dimethoxy-9*H*-xanthen-9-one (**28**).

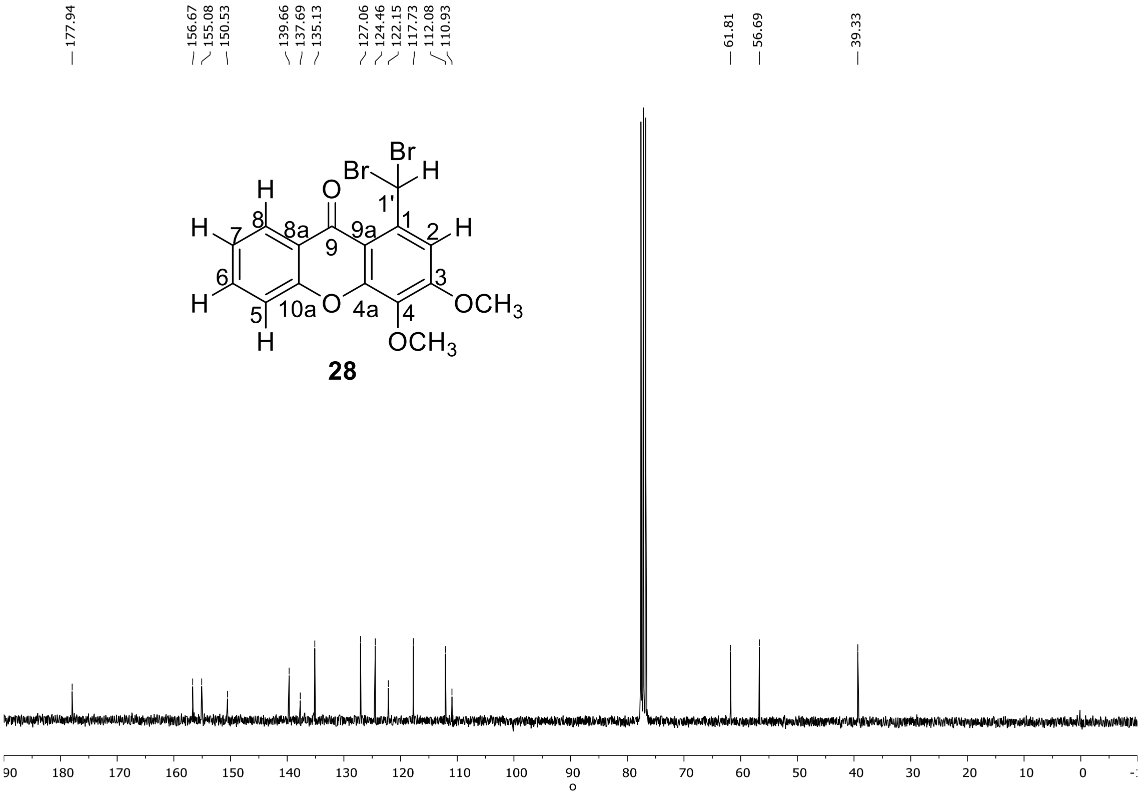

Figure S21. ^{13}C-NMR spectrum of 1-(dibromomethyl)-3,4-dimethoxy-9*H*-xanthen-9-one (**28**).

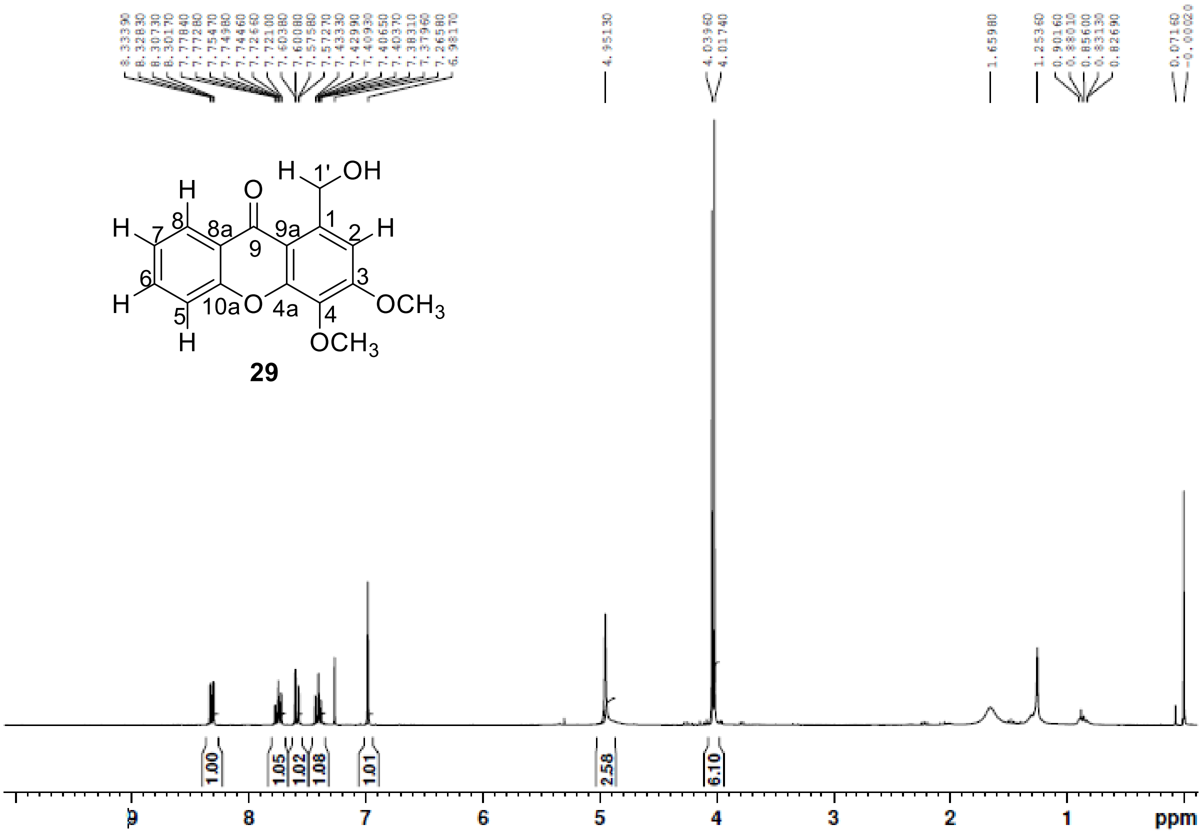

Figure S22. ^{1}H-NMR spectrum of 1-(hydroxymethyl)-3,4-dimethoxy-9*H*-xanthen-9-one (**29**).

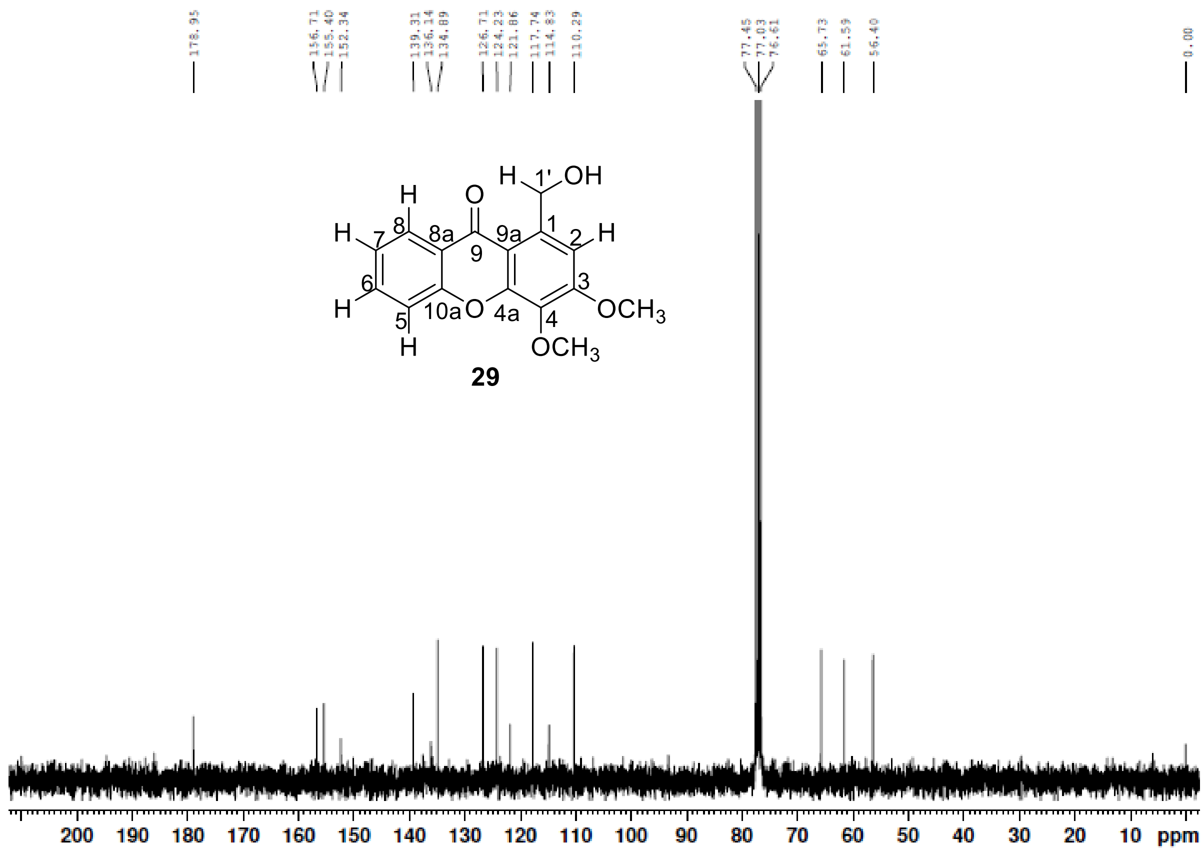

Figure S23. ^{13}C-NMR spectrum of 1-(hydroxymethyl)-3,4-dimethoxy-9*H*-xanthen-9-one (**29**).

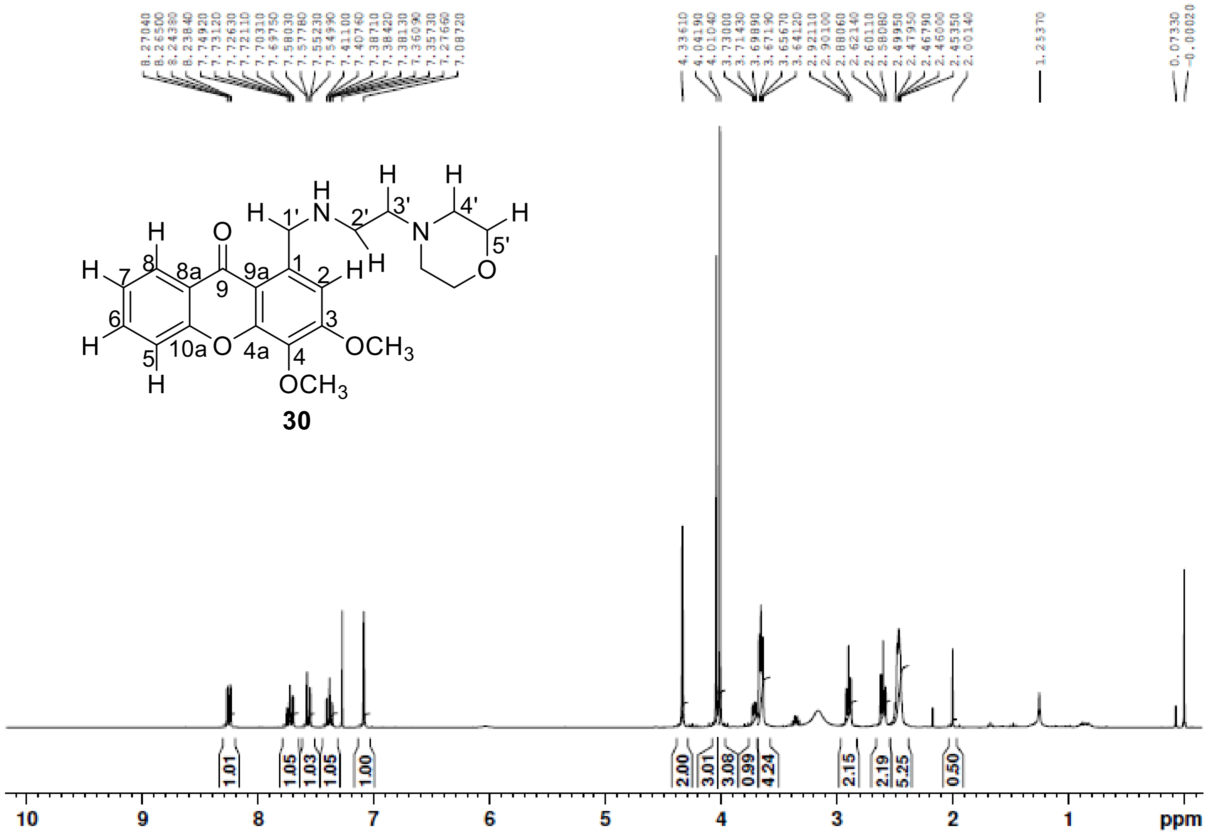

Figure S24. ^{1}H-NMR spectrum of 3,4-dimethoxy-1-(((2-morpholinoethyl)amino)methyl)-9*H*-xanthen-9-one (**30**).

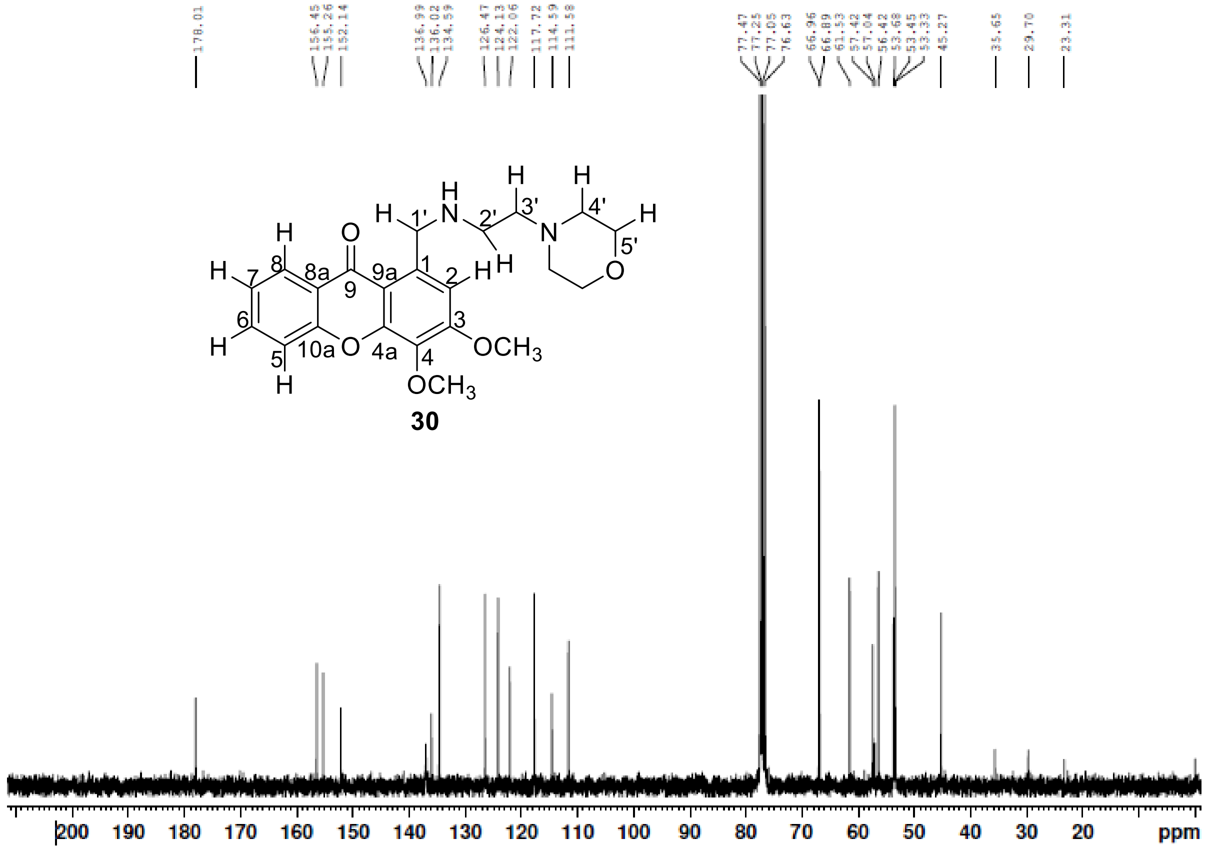

Figure S25. ^{13}C-NMR spectrum of 3,4-dimethoxy-1-(((2-morpholinoethyl)amino)methyl)-9*H*-xanthen-9-one (**30**).

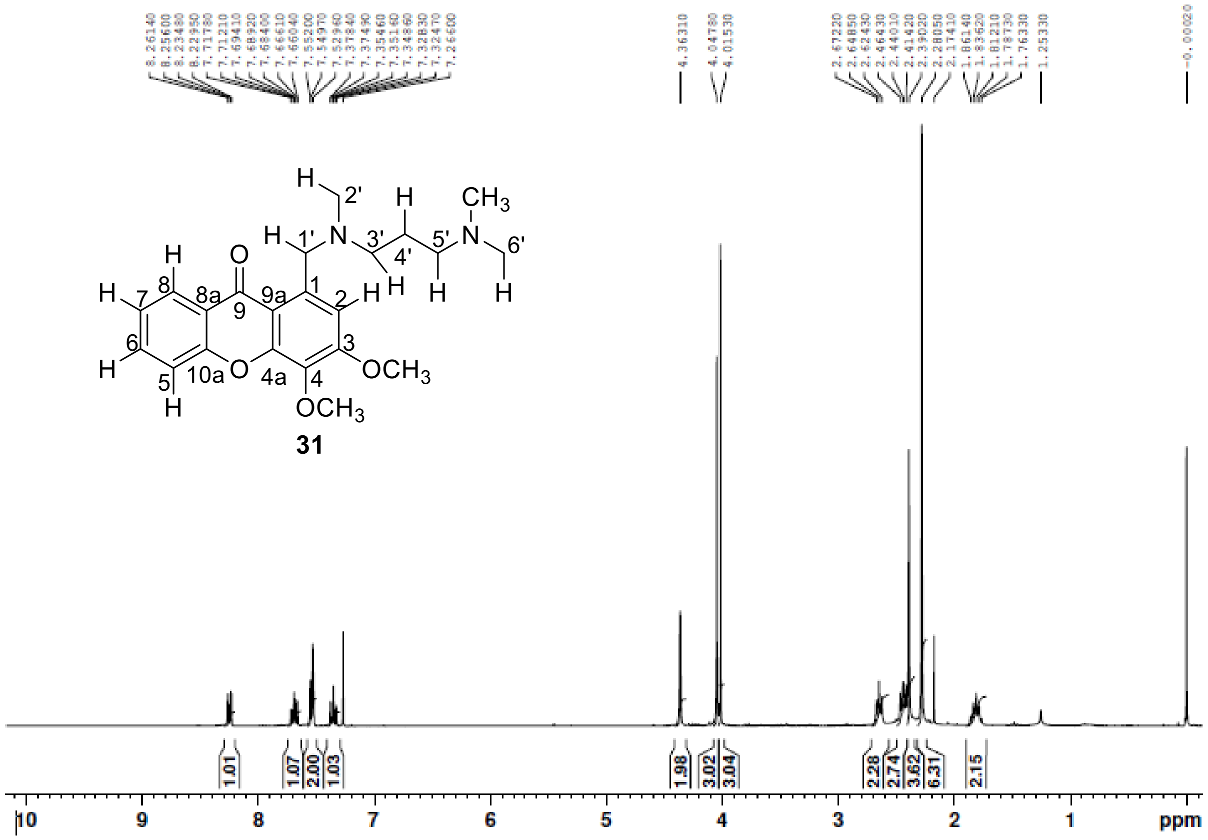

Figure S26. ^{1}H-NMR spectrum of 1-(((3-(dimethylamino)propyl)(methyl)amino)methyl)-3,4-dimethoxy-9*H*-xanthen-9-one (**31**).

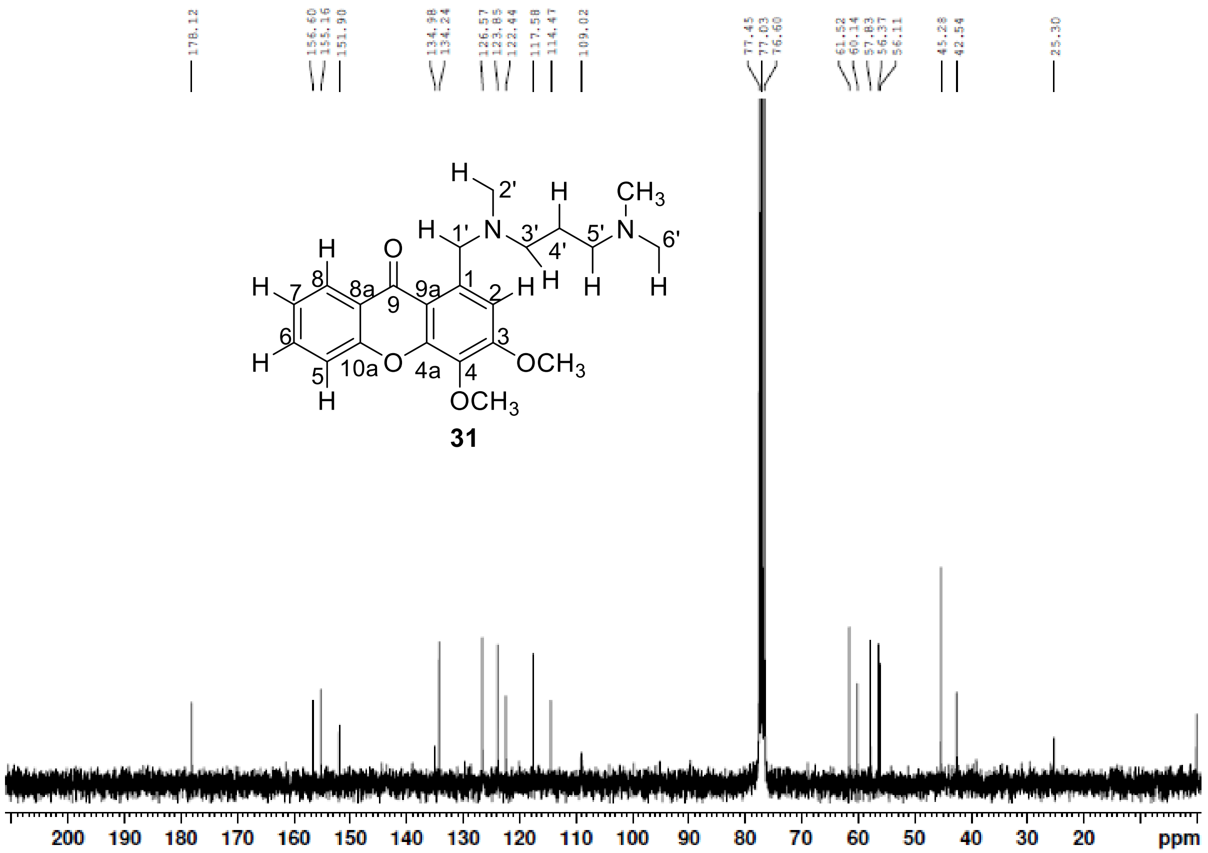

Figure S27. ^{13}C-NMR spectrum of 1-(((3-(dimethylamino)propyl)(methyl)amino)methyl)-3,4-dimethoxy-9*H*-xanthen-9-one (**31**).

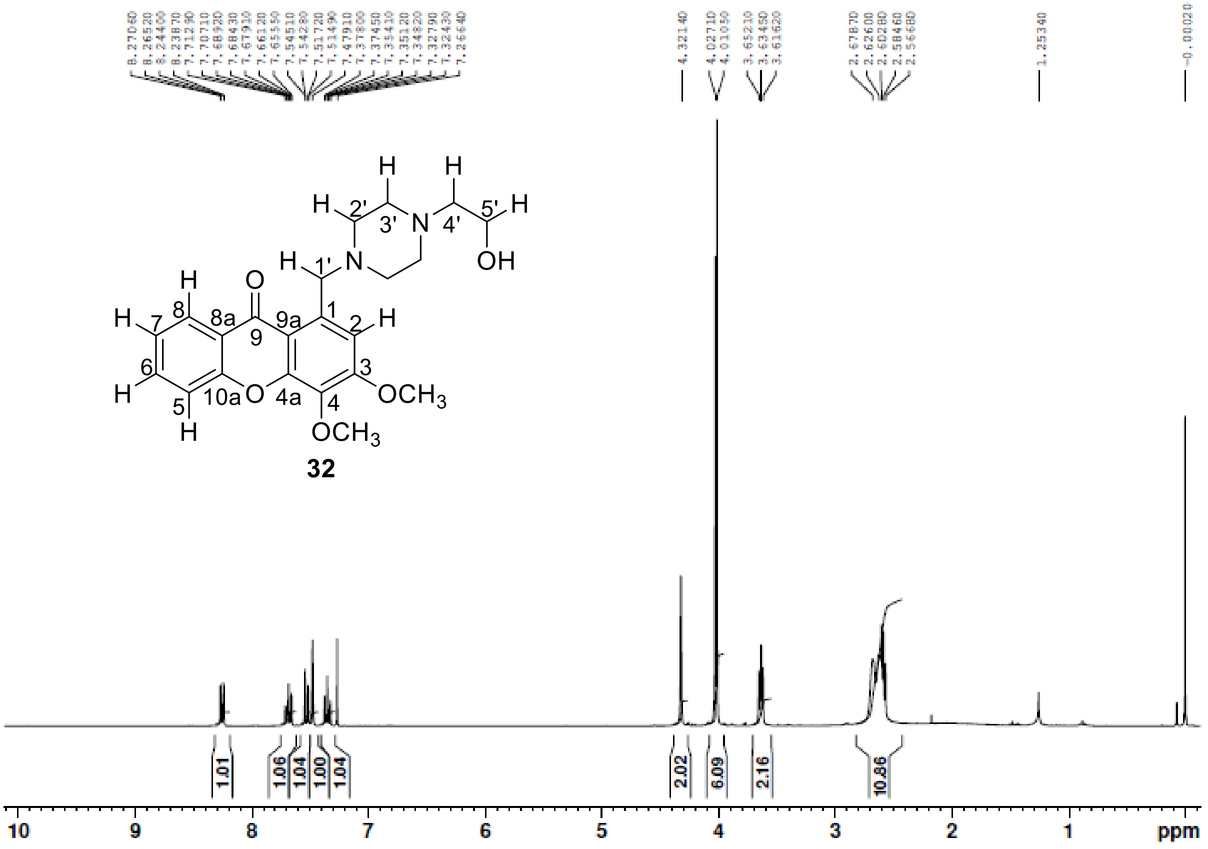

Figure S28. ^{1}H-NMR spectrum of 1-((4-(2-hydroxyethyl)piperazin-1-yl)methyl)-3,4-dimethoxy-9*H*-xanthen-9-one (**32**).

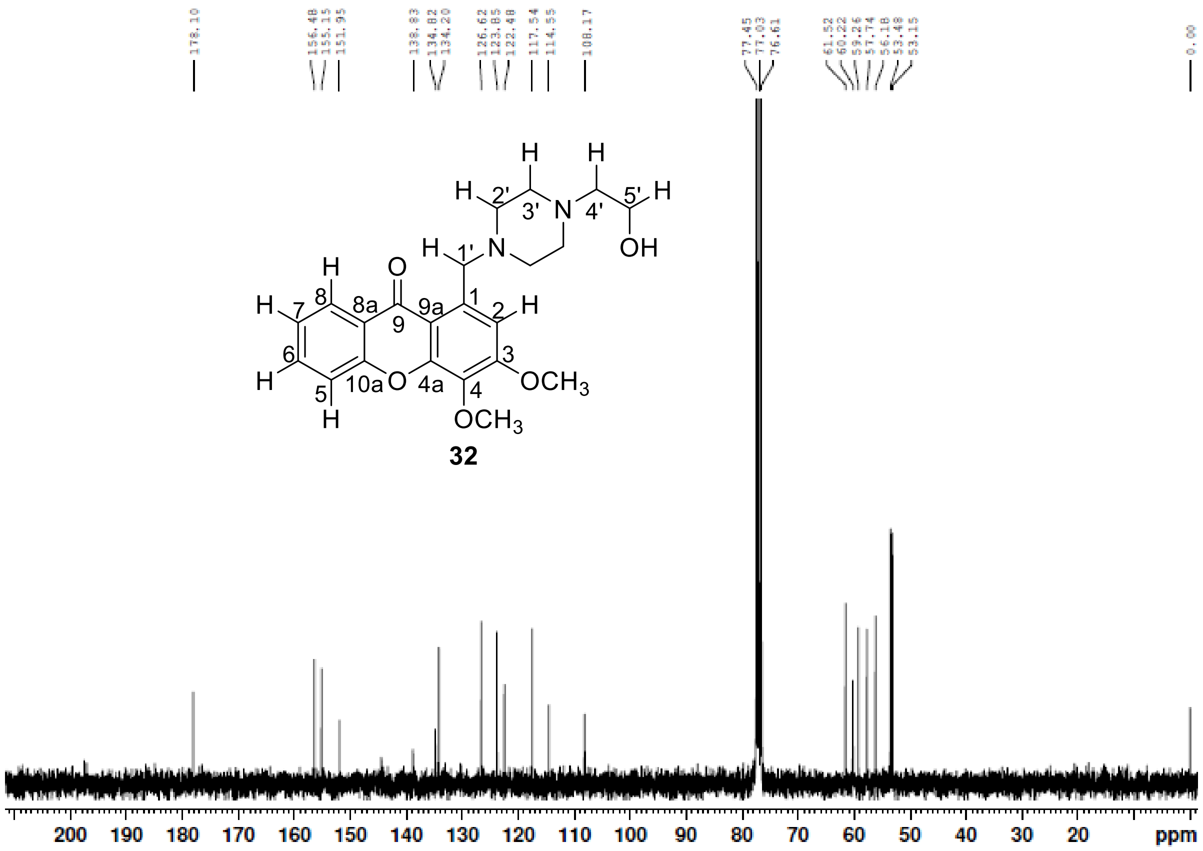

Figure S29. ^{13}C-NMR spectrum of 1-((4-(2-hydroxyethyl)piperazin-1-yl)methyl)-3,4-dimethoxy-9*H*-xanthen-9-one (**32**).

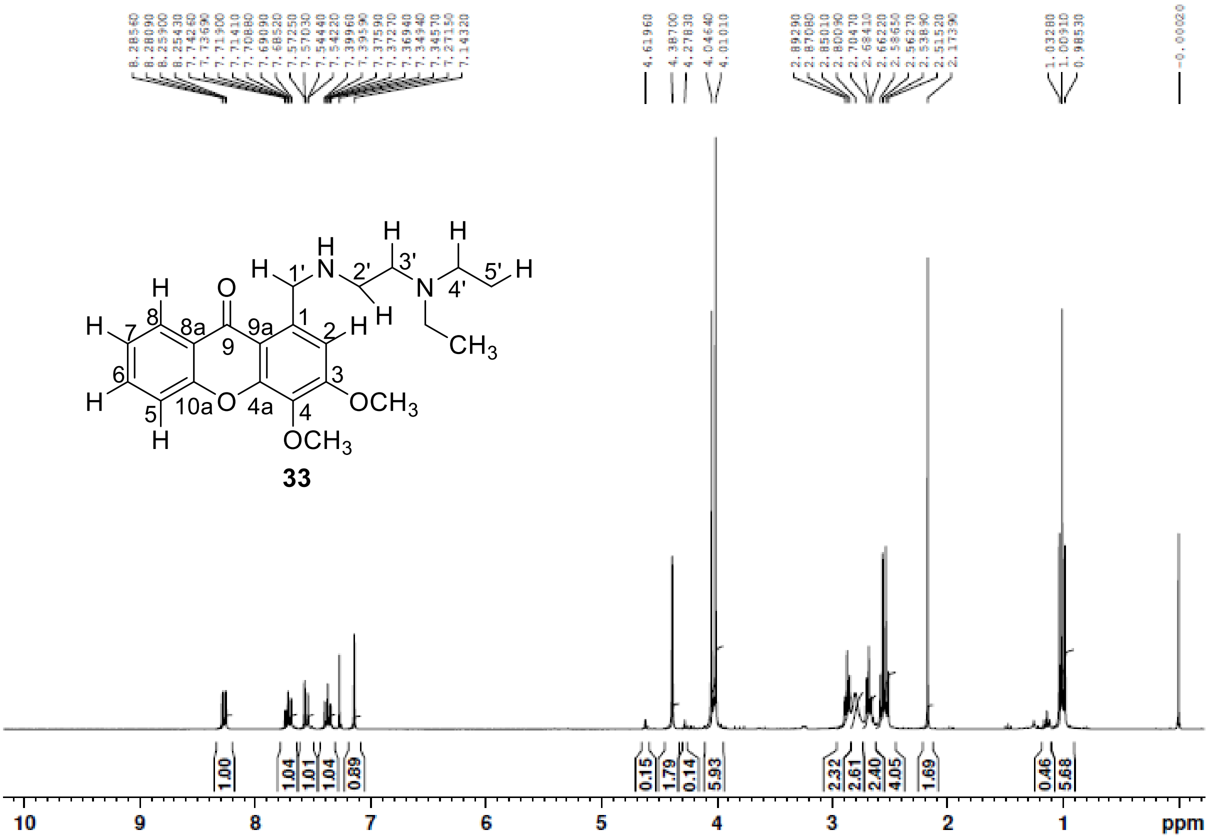

Figure S30. ^{1}H-NMR spectrum of 1-(((2-(diethylamino)ethyl)amino)methyl)-3,4-dimethoxy-9*H*-xanthen-9-one (**33**).

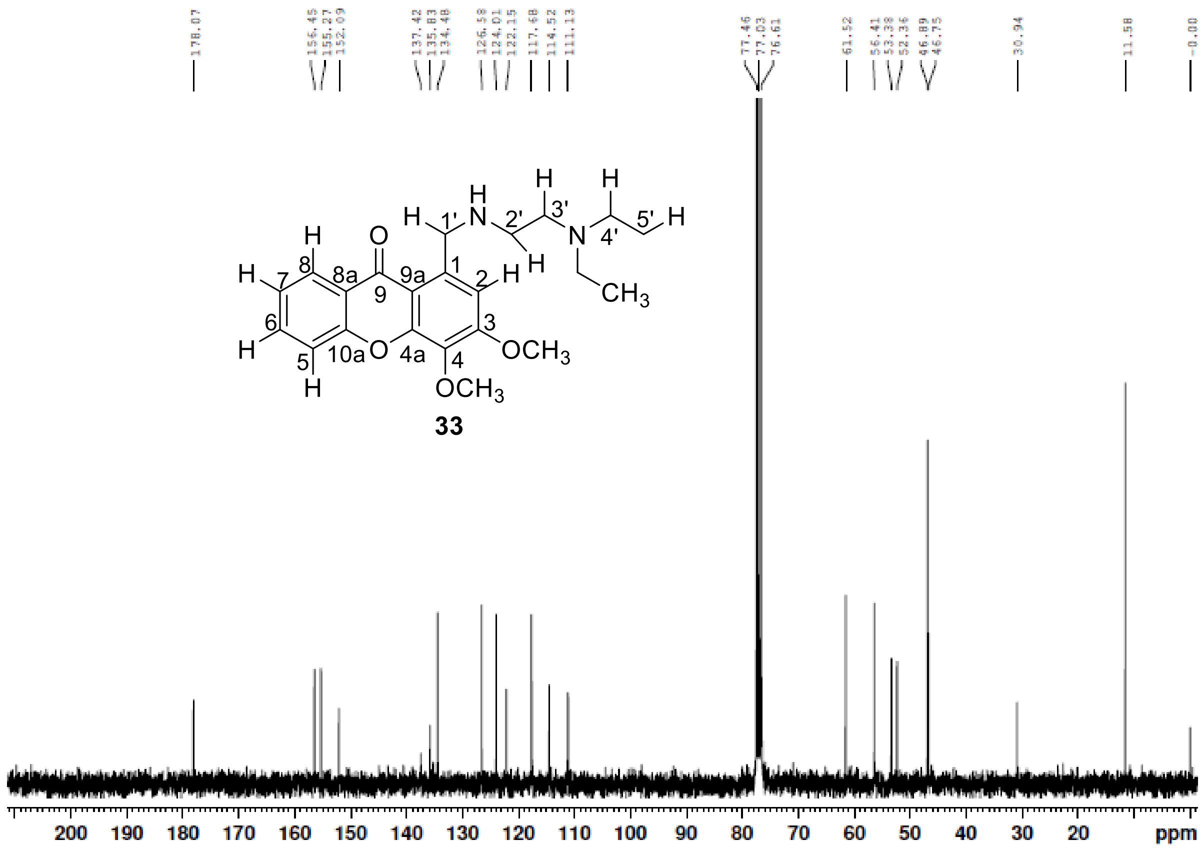

Figure S31. ^{13}C-NMR spectrum of 1-(((2-(diethylamino)ethyl)amino)methyl)-3,4-dimethoxy-9*H*-xanthen-9-one (**33**).

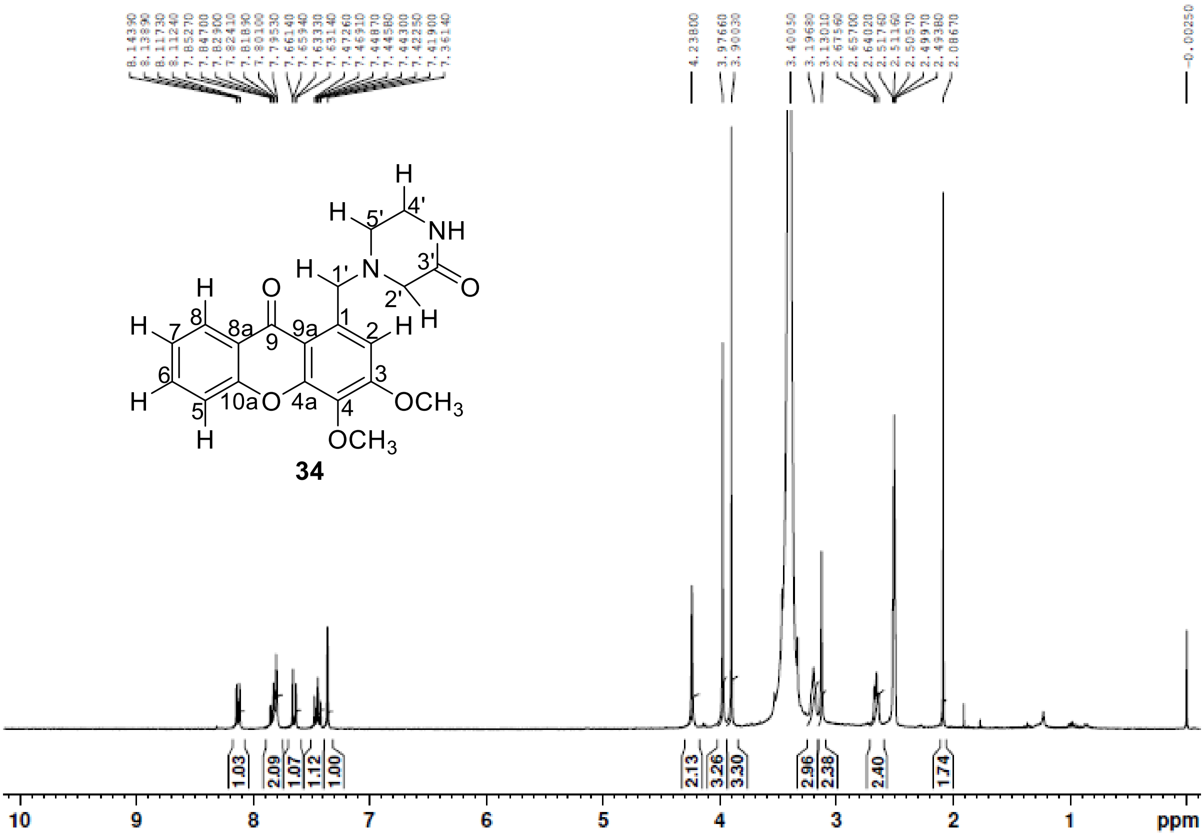

Figure S32. ^{1}H-NMR spectrum of 4-((3,4-dimethoxy-9-oxo-9*H*-xanthen-1-yl)methyl)piperazin-2-one (**34**).

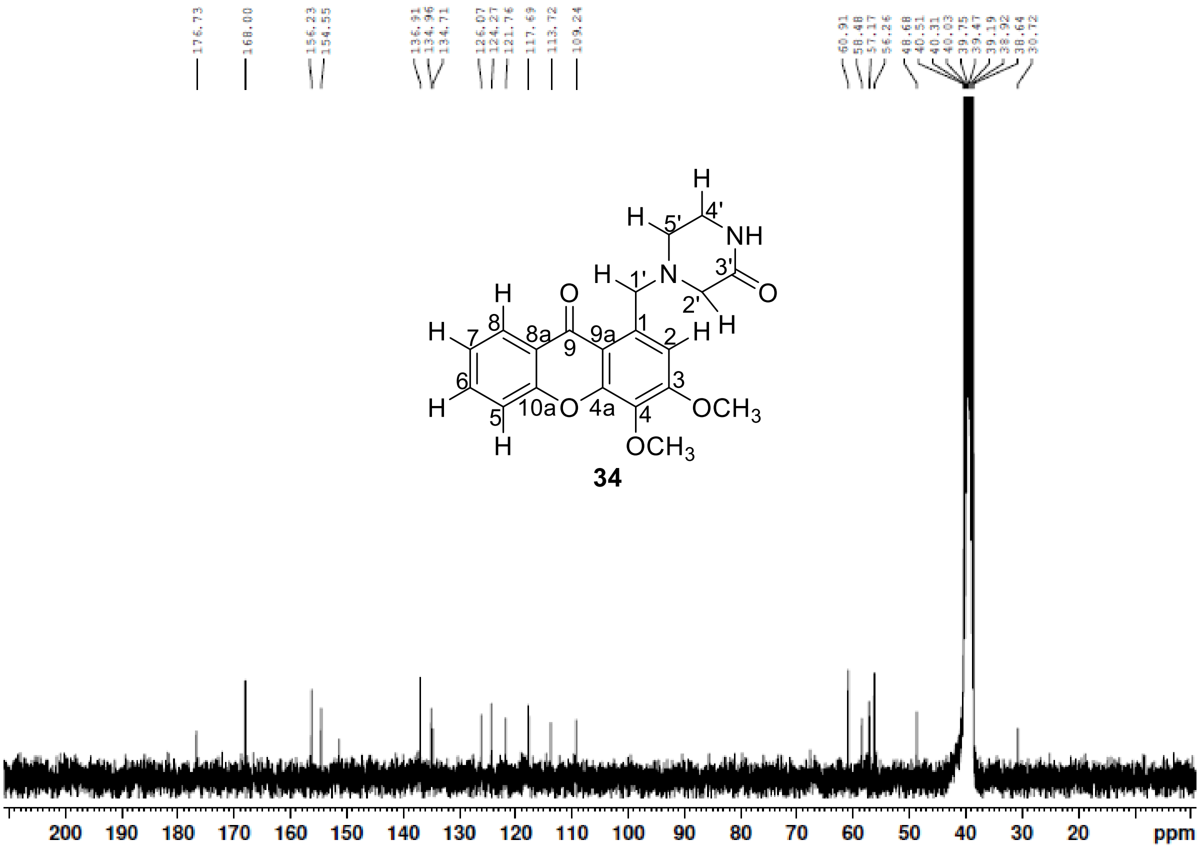

Figure S33. ^{13}C-NMR spectrum of 4-((3,4-dimethoxy-9-oxo-9*H*-xanthen-1-yl)methyl)piperazin-2-one (**34**).

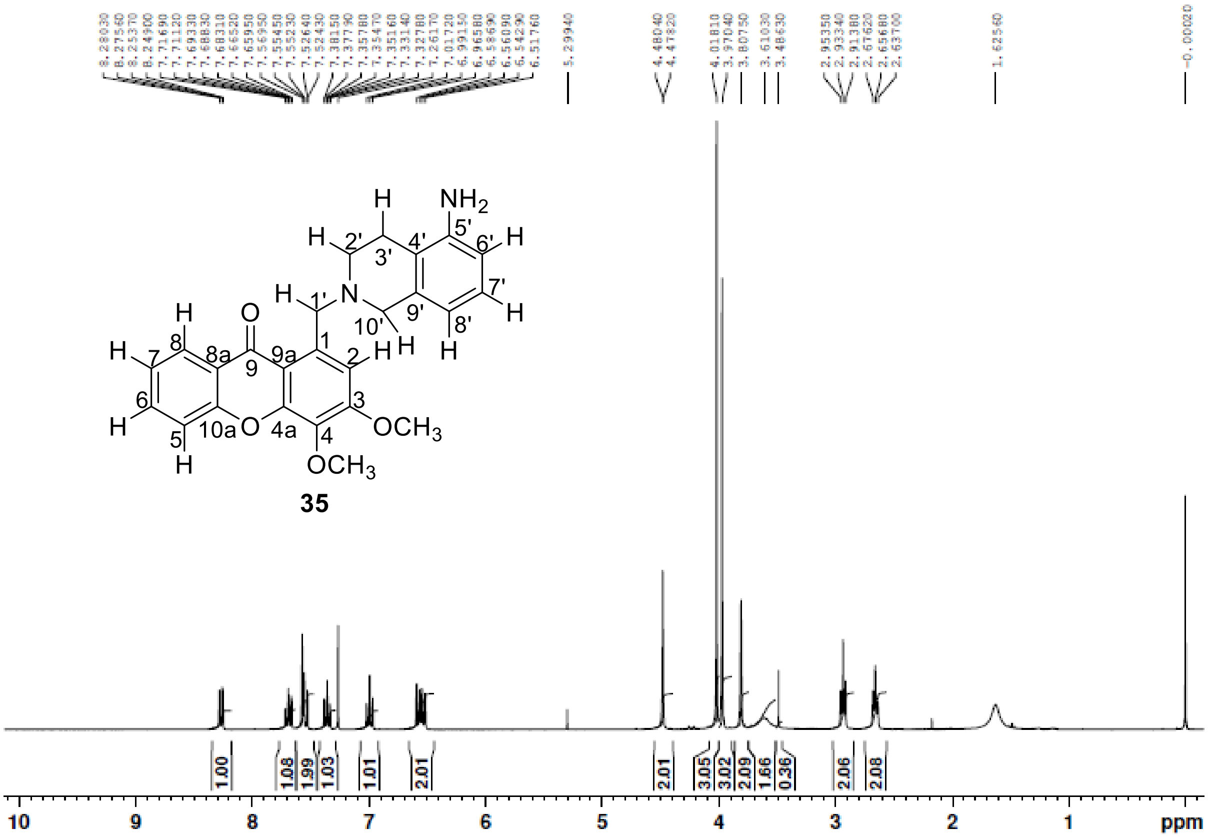

Figure S34. ^{1}H-NMR spectrum of 1-((5-amino-3,4-dihydroisoquinolin-2(1*H*)-yl)methyl)-3,4-dimethoxy-9*H*-xanthen-9-one (**35**).

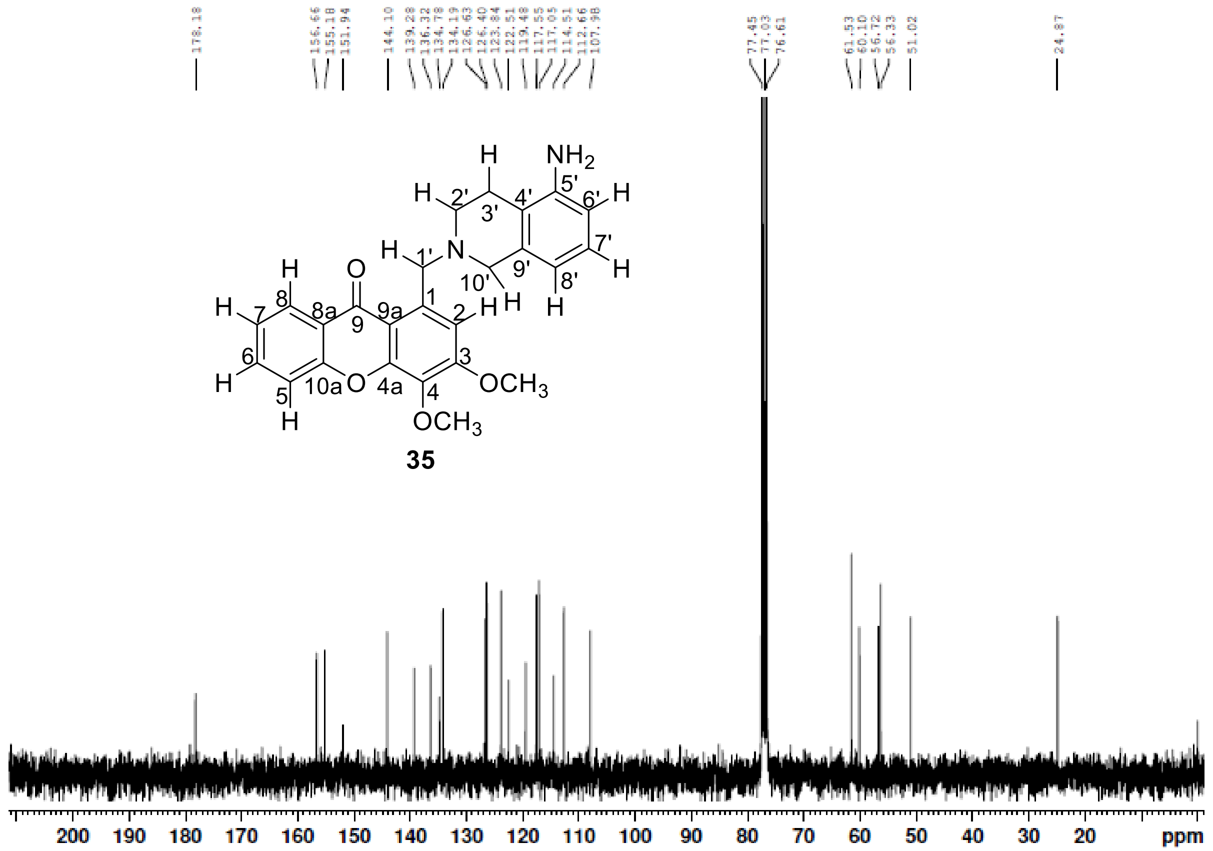

Figure S35. ^{13}C-NMR spectrum of 1-((5-amino-3,4-dihydroisoquinolin-2(1*H*)-yl)methyl)-3,4-dimethoxy-9*H*-xanthen-9-one (**35**).

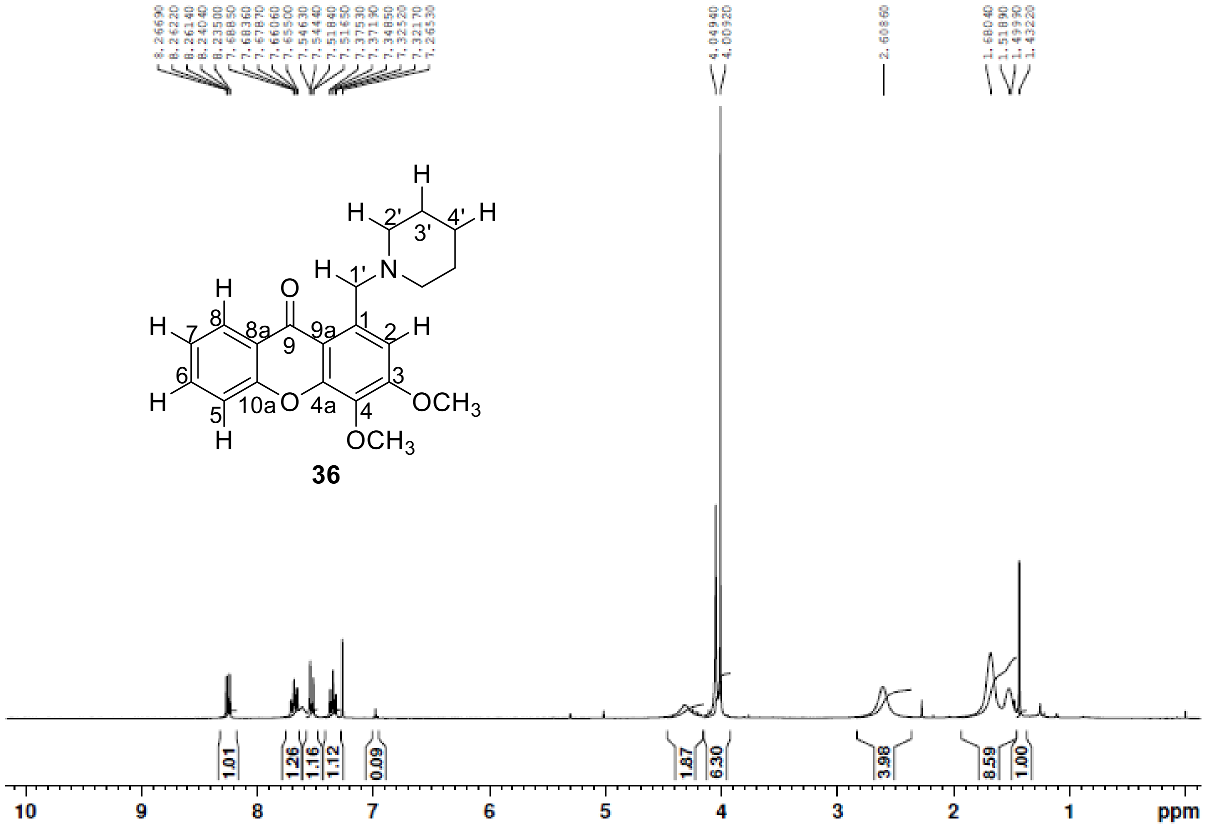

Figure S36. ^{1}H-NMR spectrum of 3,4-dimethoxy-1-(piperidin-1-ylmethyl)-9*H*-xanthen-9-one (**36**).

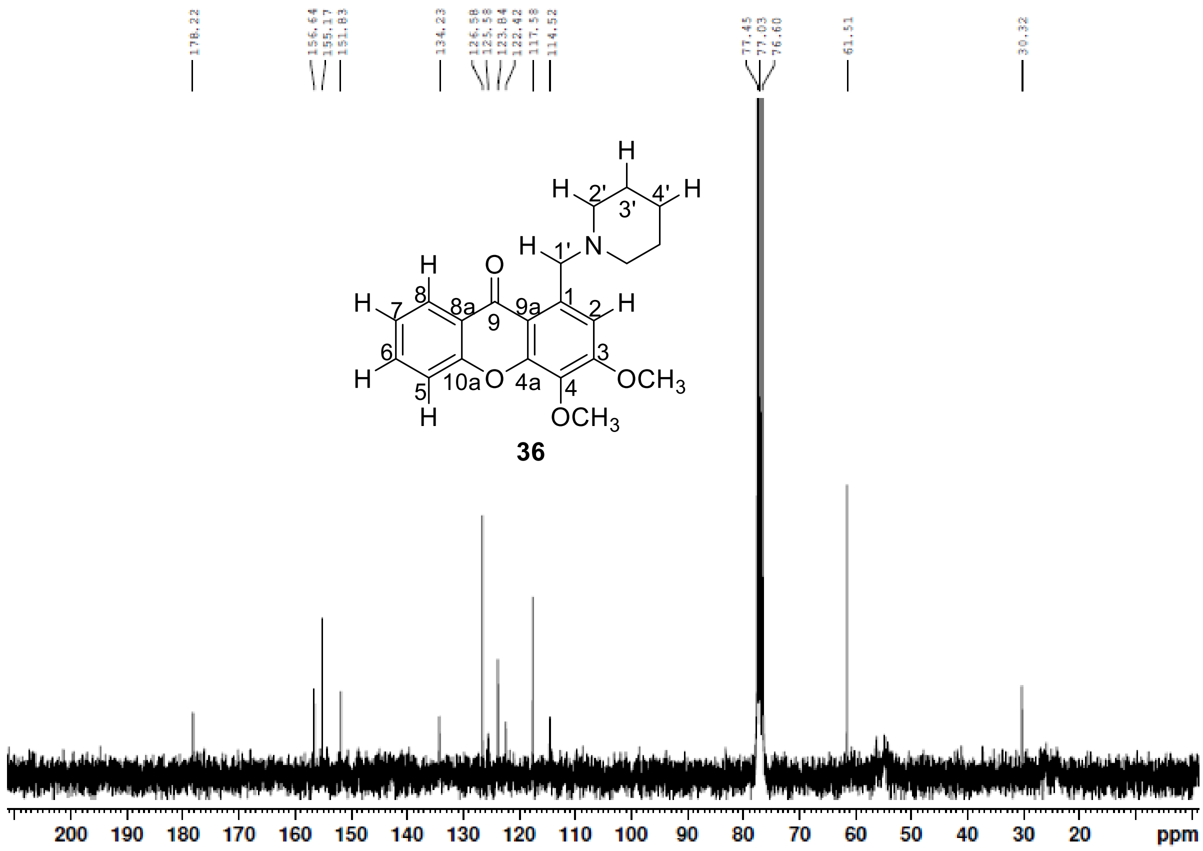

Figure S37. ^{13}C-NMR spectrum of 3,4-dimethoxy-1-(piperidin-1-ylmethyl)-9*H*-xanthen-9-one (**36**).

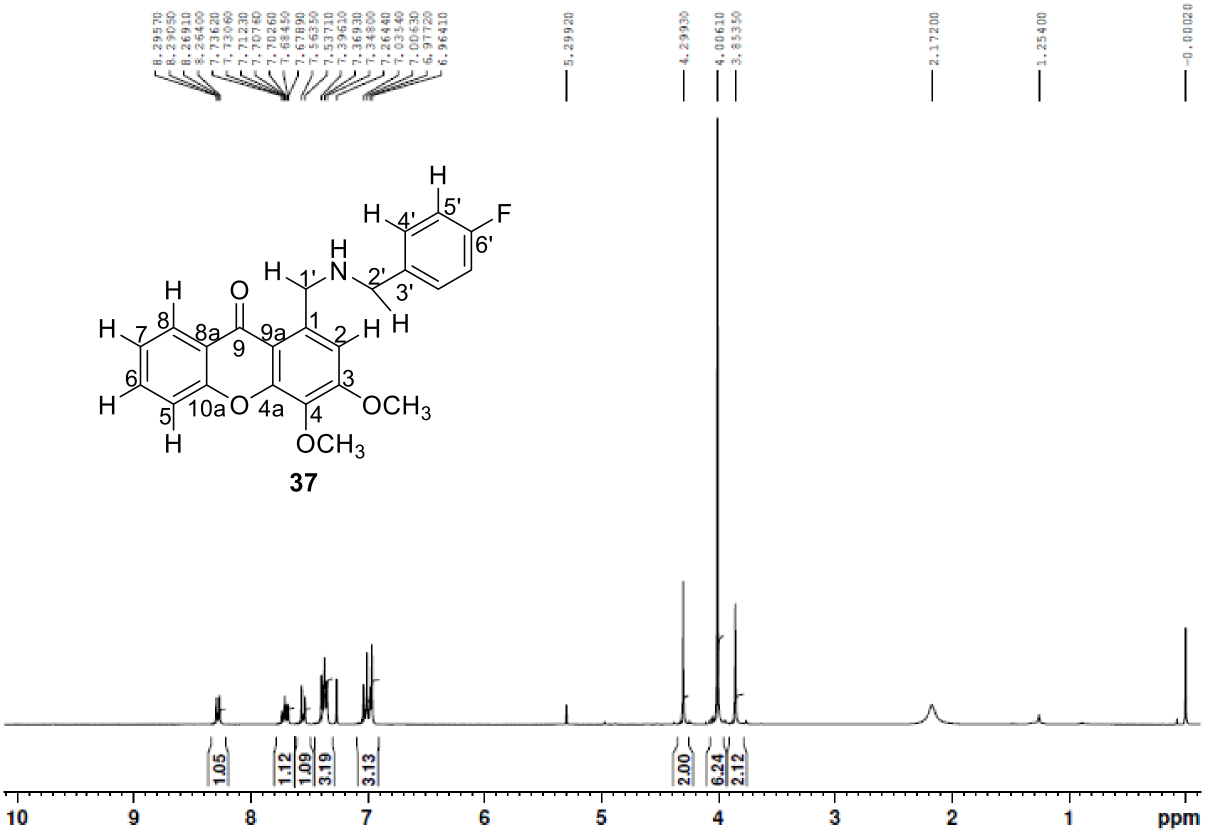

Figure S38. ^{1}H-NMR spectrum of 1-(((4-fluorobenzyl)amino)methyl)-3,4-dimethoxy-9*H*-xanthen-9-one (**37**).

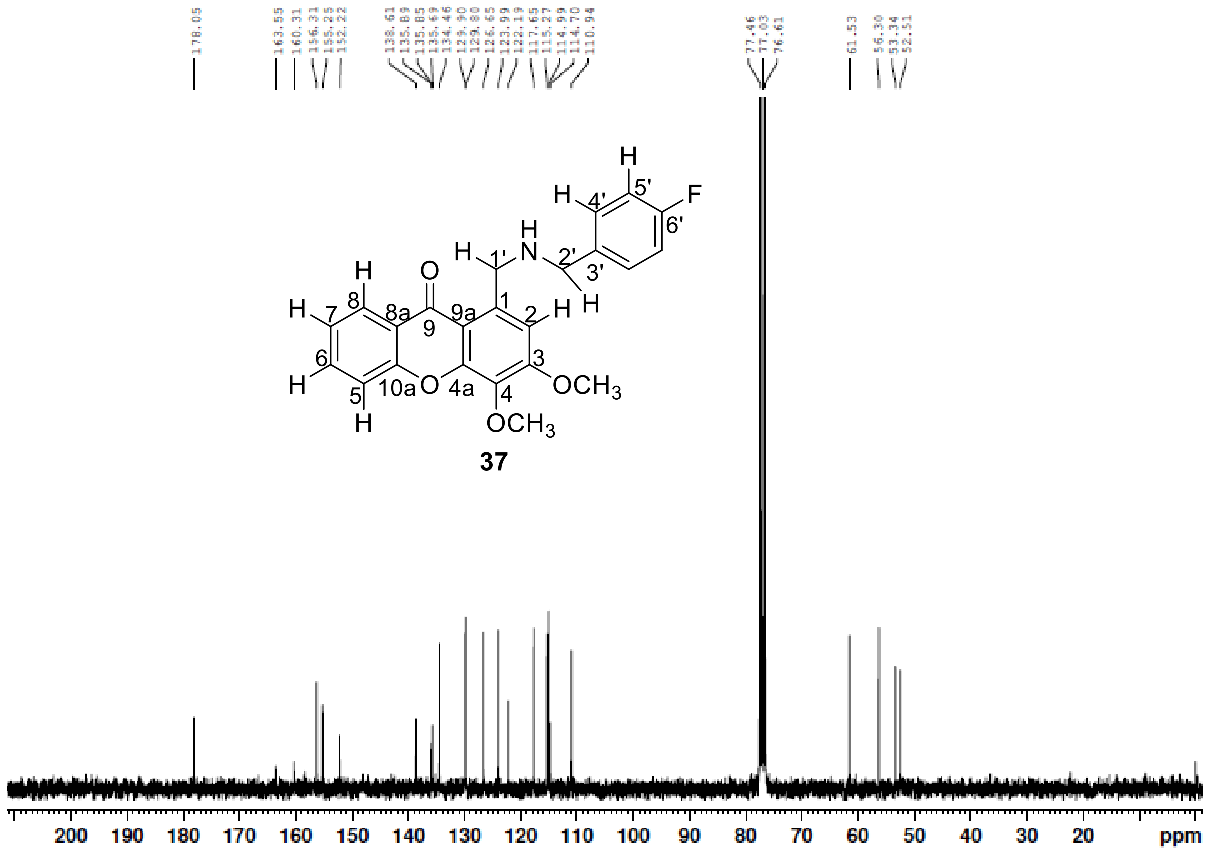

Figure S39. ^{13}C-NMR spectrum of 1-(((4-fluorobenzyl)amino)methyl)-3,4-dimethoxy-9*H*-xanthen-9-one (**37**).

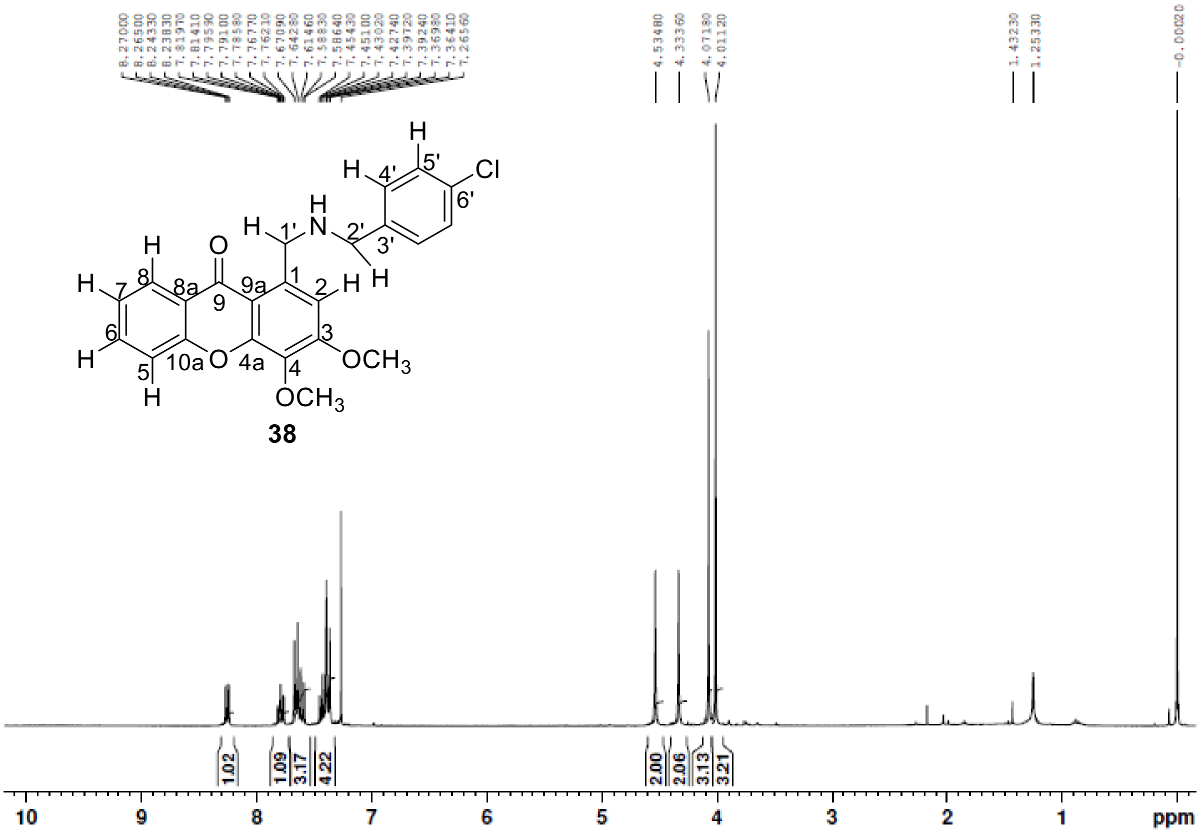

Figure S40. ^{1}H-NMR spectrum of 1-(((4-chlorobenzyl)amino)methyl)-3,4-dimethoxy-9*H*-xanthen-9-one (**38**).

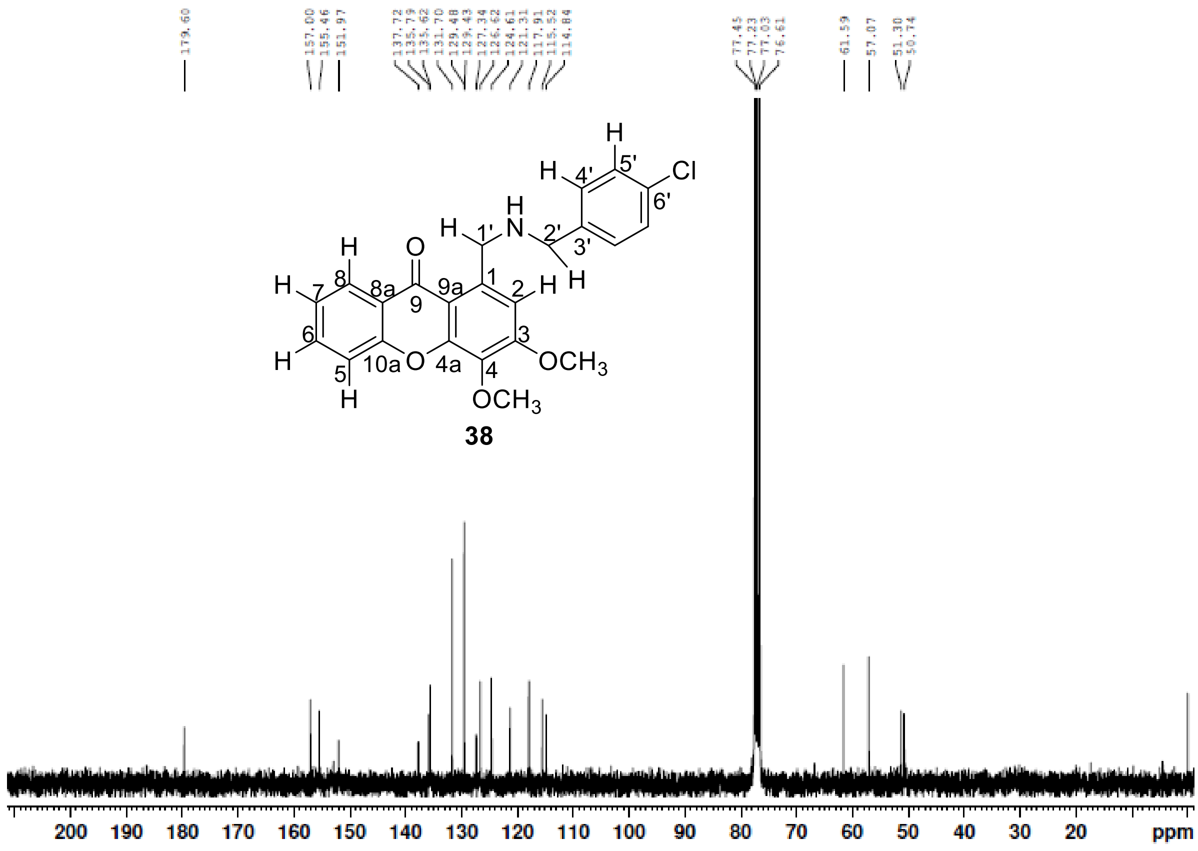

Figure S41. ^{13}C-NMR spectrum of 1-(((4-chlorobenzyl)amino)methyl)-3,4-dimethoxy-9*H*-xanthen-9-one (**38**).

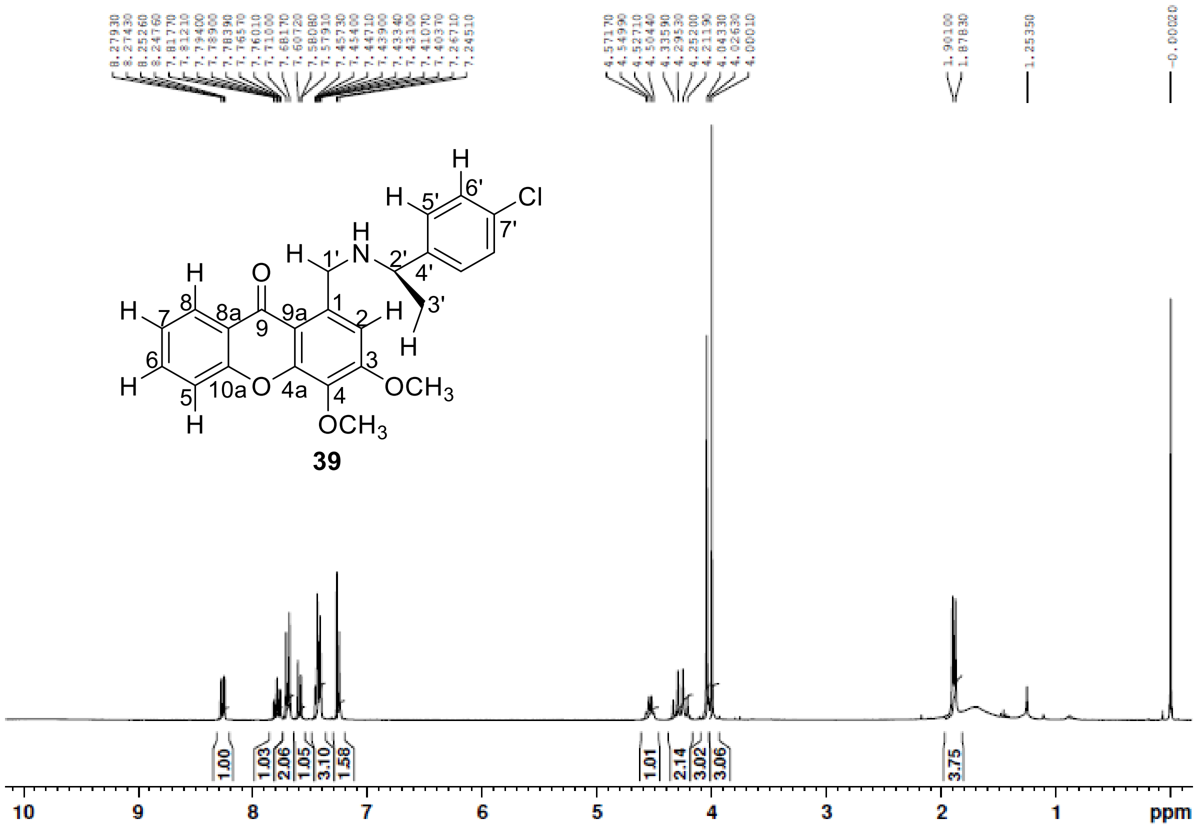

Figure S42. ^{1}H-NMR spectrum of (*R*)-1-(((1-(4-chlorophenyl)ethyl)amino)methyl)-3,4-dimethoxy-9*H*-xanthen-9-one (**39**).

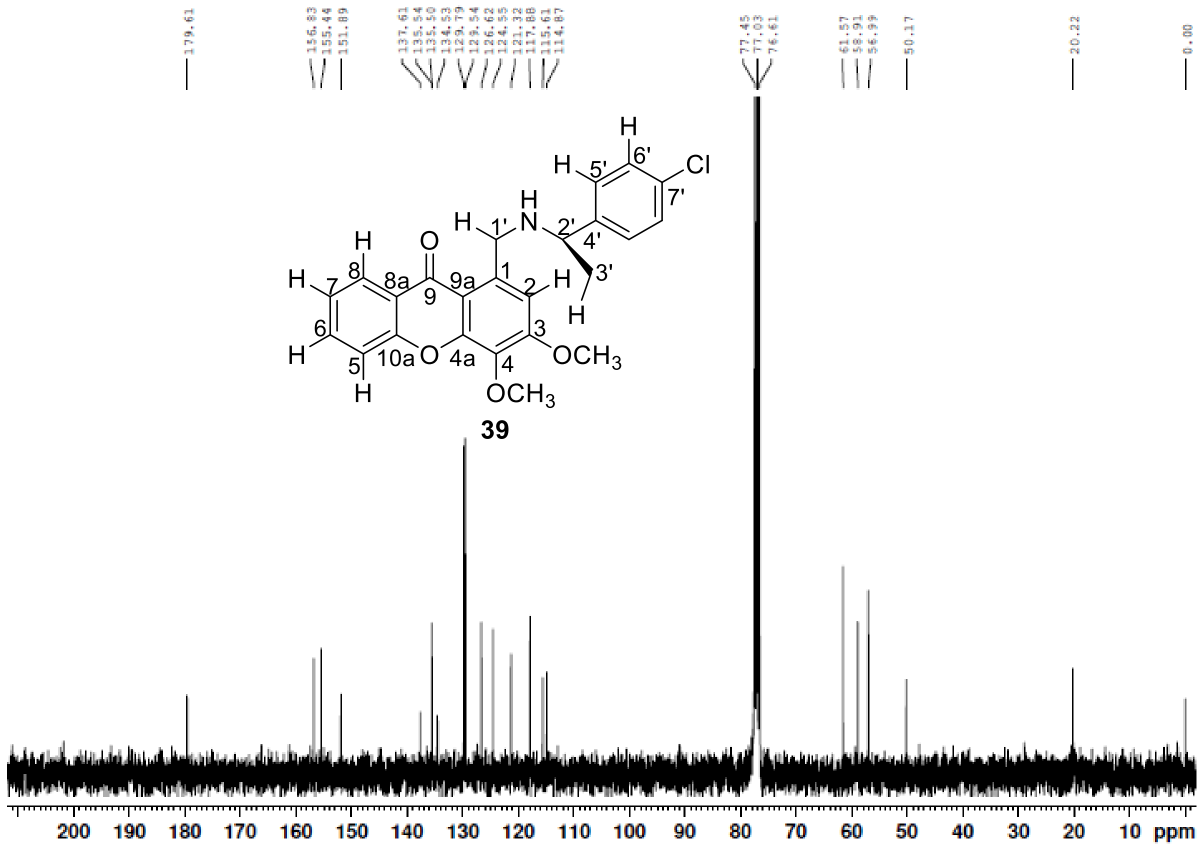

Figure S43. ^{13}C-NMR spectrum of (*R*)-1-(((1-(4-chlorophenyl)ethyl)amino)methyl)-3,4-dimethoxy-9*H*-xanthen-9-one (**39**).

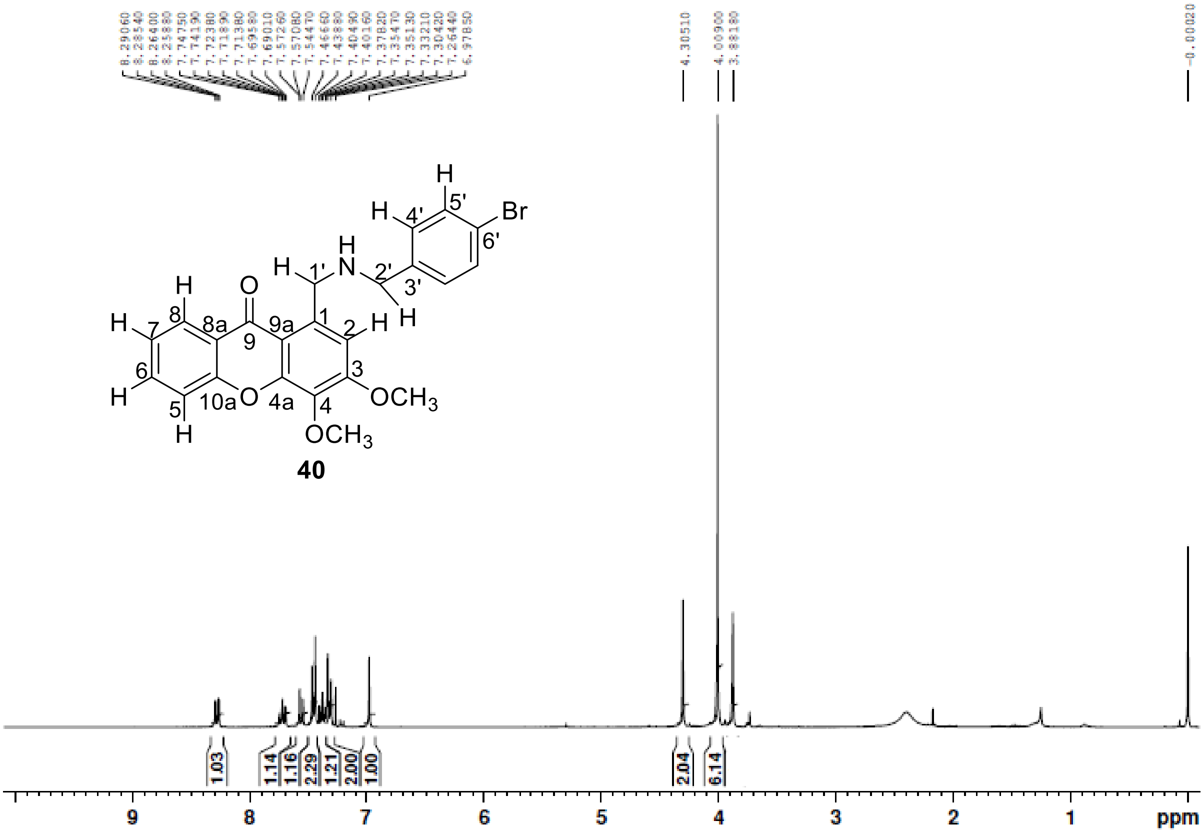

Figure S44. ^{1}H-NMR spectrum of 1-(((4-bromobenzyl)amino)methyl)-3,4-dimethoxy-9*H*-xanthen-9-one (**40**).

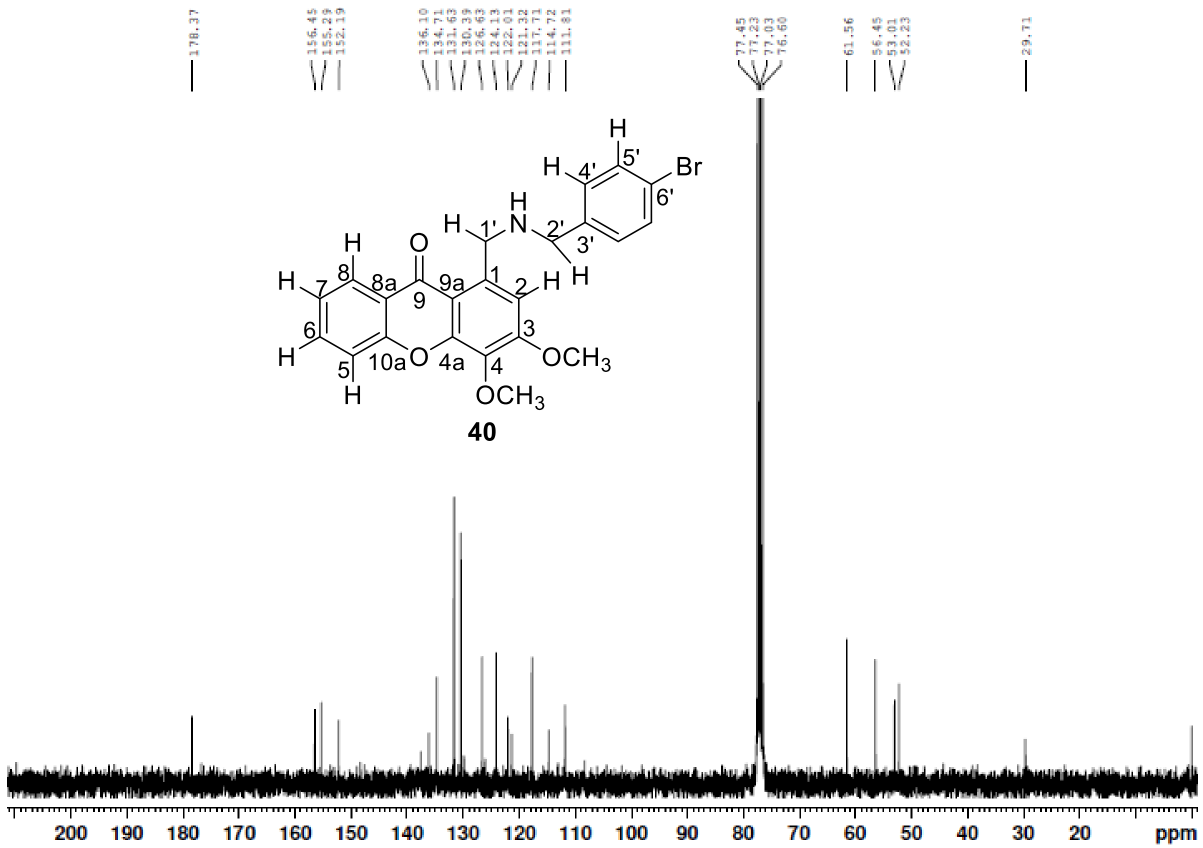

Figure S45. ^{13}C-NMR spectrum of 1-(((4-bromobenzyl)amino)methyl)-3,4-dimethoxy-9*H*-xanthen-9-one (**40**).
